# A Population-Based Descriptive Atlas of Invasive Pneumococcal Strains Recovered Within the U.S. During 2015–2016

**DOI:** 10.3389/fmicb.2018.02670

**Published:** 2018-11-19

**Authors:** Bernard Beall, Sopio Chochua, Robert E. Gertz, Yuan Li, Zhongya Li, Lesley McGee, Benjamin J. Metcalf, Jessica Ricaldi, Theresa Tran, Hollis Walker, Tamara Pilishvili

**Affiliations:** Respiratory Diseases Branch, Centers for Disease Control and Prevention, Atlanta, GA, United States

**Keywords:** conjugate vaccine, pneumococcal, clonal complex (CC), serotype diversity, serotype distributions, antibiotic resistance

## Abstract

Invasive pneumococcal disease (IPD) has greatly decreased since implementation in the U.S. of the 7 valent conjugate vaccine (PCV7) in 2000 and 13 valent conjugate vaccine (PCV13) in 2010. We used whole genome sequencing (WGS) to predict phenotypic traits (serotypes, antimicrobial phenotypes, and pilus determinants) and determine multilocus genotypes from 5334 isolates (~90% of cases) recovered during 2015–2016 through Active Bacterial Core surveillance. We identified 44 serotypes; 26 accounted for 98% of the isolates. PCV13 serotypes (inclusive of serotype 6C) accounted for 1503 (28.2%) isolates, with serotype 3 most common (657/5334, 12.3%), while serotypes 1 and 5 were undetected. Of 305 isolates from children <5 yrs, 60 (19.7%) were of PCV13 serotypes 19A, 19F, 3, 6B, and 23F (58/60 were 19A, 19F, or 3). We quantitated MLST-based lineages first detected during the post-PCV era (since 2002) that potentially arose through serotype-switching. The 7 predominant emergent post-PCV strain complexes included 23B/CC338, 15BC/CC3280, 19A/CC244, 4/CC439, 15A/CC156, 35B/CC156, and 15BC/CC156. These strains accounted for 332 isolates (6.2% of total) and were more frequently observed in children <5 yrs (17.7%; 54/305). Fifty-seven categories of recently emerged (in the post PCV7 period) putative serotype-switch variants were identified, accounting for 402 isolates. Many of these putative switch variants represented newly emerged resistant strains. Penicillin-nonsusceptibility (MICs > 0.12 μg/ml) was found among 22.4% (1193/5334) isolates, with higher penicillin MICs (2–8 μg/ml) found in 8.0% (425/5334) of isolates that were primarily (372/425, 87.5%) serotypes 35B and 19A. Most (792/1193, 66.4%) penicillin-nonsusceptible isolates were macrolide-resistant, 410 (34.4%) of which were *erm* gene positive and clindamycin-resistant. The proportion of macrolide-resistant isolates increased with increasing penicillin MICs; even isolates with reduced penicillin susceptibility (MIC = 0.06 μg/ml) were much more likely to be macrolide-resistant than basally penicillin-susceptible isolates (MIC < 0.03 μg/ml). The contribution of recombination to strain diversification was assessed through quantitating 35B/CC558-specific bioinformatic pipeline features among non-CC558 CCs and determining the sizes of gene replacements. Although IPD has decreased greatly and stabilized in the post-PCV13 era, the species continually generates recombinants that adapt to selective pressures exerted by vaccines and antimicrobials. These data serve as a baseline for monitoring future changes within each invasive serotype.

## Introduction

The two major emphases of IPD strain surveillance, identification of serotype distributions and antimicrobial resistance phenotype, have not changed over several decades. The distributions of these two basic pneumococcal strain features informs strategic formulation of next-generation vaccines, evaluation of current vaccines, and establishment of appropriate antibiotic usage for clinical cases. Pneumococcal conjugate vaccines (PCVs) are very effective in reducing incidence of PCV-type IPD (Pilishvili et al., 2010; Moore et al., 2015) and pneumonia in children (Nelson et al., 2008; Olarte et al., 2017a), and widespread use in children has substantially reduced rates of IPD and pneumonia among adults (Griffin et al., 2013; Simonsen et al., 2014). PCVs prevent acquisition of vaccine-type pneumococcal carriage in children which serves to reduce transmission to other children and adults. Pneumococcal conjugate vaccines have not only greatly reduced IPD, but have preferentially targeted antimicrobial-resistant strains. Introductions of both PCV7 (in 2000) and PCV13 (in 2010) both served to dramatically decrease IPD caused by antibiotic-resistant strains in all ages, particularly strains resistant to penicillins (Kyaw et al., 2006; Tomczyk et al., 2016).

Neither of the past two PCVs had entirely predictable effects. While PCV7 greatly decreased overall disease through reduction of PCV7-type disease, the dramatic emergence of the highly resistant 19A/ST320 was an unexpected negative event. Besides impacting the emergence of pre-existing uncommon strains (for example 19A/ST320 before PCV7 implementation), circumstantial data indicates that serotype-switch strains arising through recombinational replacement of the *cps* locus can be amplified through PCV selective pressure. For example, the 19A/ST695 variant resulting from a PCV7 serotype 4 to non-PCV7 type 19A switch, was first detected in the post-PCV7 period and spread throughout the country to become the 2nd most frequent 19A strain complex (Pai et al., 2005b; Brueggemann et al., 2007; Beall et al., 2011; Golubchik et al., 2012). Similarly, while PCV13 has dramatically decreased PCV serotype IPD, the multi-resistant 35B/ST156 variant was detected soon after PCV13 implementation (Metcalf et al., 2016b; Olarte et al., 2017b) and quickly became the 2nd leading 35B strain complex (Chochua et al., 2017). It will not be surprising if detected recombination events involving non-PCV serotypes continue to increase since carriage of these serotypes within the principal nasopharyngeal reservoir in children has substantially increased in the post PCV7 and PCV13 periods (Sharma et al., 2013; Desai et al., 2015).

Here we reveal distributions of serotypes and key antimicrobial phenotypes for invasive disease isolates identified through routine surveillance in 2015–2016. In addition, we depict basic strain structures of each individual serotype and quantitate strain complexes that have only become apparent within the post-PCV period.

## Materials and methods

### IPD isolates

All 5334 isolates characterized were identified through CDC's Active Bacterial Core surveillance (ABCs) during 2015–2016. ABCs is an active population and laboratory-based system which covers a population of approximately 32.2 million individuals. ABCs areas, methods, and key surveillance data through 2016 are described at https://www.cdc.gov/abcs/reports-findings/survreports/spneu16.html. WGS accessions are only provided for the 5212 isolates that yielded high quality sequencing metrics (sTable 1).

### Genomic sequencing

Genomic DNA preparation, library construction, whole genome sequencing (WGS), and bioinformatics pipeline features have been previously described (Li et al., 2016, 2017; Metcalf et al., 2016a,b). *Streptococcus pneumoniae* strains were cultured on Trypticase soy agar supplemented with 5% sheep blood and incubated overnight at 37°C in 5% CO_2_. Genomic DNA for short-read WGS was extracted manually using a modified QIAamp DNA mini kit protocol (Qiagen, Inc., Valencia, CA, USA). Nucleic acid concentration was quantified by an Invitrogen Qubit assay (Thermo Fisher Scientific Inc., Waltham, MA, USA) and samples were sheared using a Covaris M220 ultrasonicator (Covaris, Inc., Woburn, MA, USA) programmed to generate 500-bp fragments. Libraries were constructed on the SciCloneG3 (PerkinElmer Inc., Waltham, MA, USA) using a TruSeq DNA PCR-Free HT library preparation kit with 96 dual indices (Illumina Inc., San Diego, CA, USA) and quantified by a KAPA qPCR library quantification method (Kapa Biosystems Inc., Wilmington, MA, USA). WGS was generated employing two MiSeq instruments and the MiSeq v2 500 cycle kit (Illumina Inc).

### Strain features

Strain features were predicted using our bioinformatics pipeline (Metcalf et al., 2016a,b). Strain features included capsular serotypes, multilocus sequence typing (MLST), pilus prediction, and minimum inhibitory concentrations (MICs) for antibiotics. Strain features associated with isolate identifiers and genome accession numbers are listed for all isolates in sTable 1. Of the 5334 isolates, 5212 (~98%) were judged of sufficient quality to release to the short read archive (SRA). For ~2% of isolates, critical strain parameters, when missing, were obtained through phenotypic testing and for these no WGS data is publicly available. Critical WGS assembly metrics (contig number, n50 [an indicator of average contig size], longest contig length, sum of contig lengths) are provided in sTable 1. These WGS sequence read accession (SRA) submissions are available at https://trace.ncbi.nlm.nih.gov/Traces/study/?acc=PRJNA284954&go=go.

Penicillin-resistance (penR) equates to an MIC of ≥2 μg/ml, with intermediate resistance (penI) at 0.12–1 μg/ml and reduced susceptibility at 0.06 μg/ml. All year 2015 isolates were also subjected to conventional broth dilution testing as previously described (Metcalf et al., 2016a). Year 2016 isolates recovered from Minnesota were also subjected to conventional MIC testing and serotyping, which were in close agreement with pipeline-predicted MICs and serotypes (latter with 100% concordance). Approximately 3% of remaining year 2016 isolates were subjected to conventional testing for minimum inhibitory concentrations (MICs) due to sub-optimal assemblies affecting penicillin binding protein (PBP) typing. About 0.3 % of isolates were assigned serotypes serologically.

### Genetic analyses of strains

eBURST (Feil et al., 2004) was employed using the website http://eburst.mlst.net/v3/enter_data/single/ with the number of loci set at 10. The 10 loci included the conventional multilocus sequence typing housekeeping gene fragment identifiers (provided by https://pubmlst.org/spneumoniae/) to which we added the previously described 3 digit PBP type (Metcalf et al., 2016b). eBURST complexes consisted of isolates sharing at least 5 of the 7 housekeeping loci with other member(s) of the set.

For phylogenetic analysis of strain sets, a reference sequence was selected for the major ST from the year 2015 dataset. Snippy (https://github.com/tseemann/snippy) was used to read map genomes against the corresponding reference sequence and to build a core SNP alignment. Isolates that had under 90% coverage of the reference sequence were excluded from the analysis. Trees were built using RAxML with ascertainment correction. PlotTree (https://github.com/katholt/plotTree) was used for the visualization of metadata in trees.

Recombinant region sizes within individual strains exhibiting typing (MLST, *cps*, PBP subtype) features characteristic of 35B/CC558 strains were measured through comparison with the 35B/ST558 reference sequence from isolate 20154871 (sTable 1). Contiguous regions of > 1,000 bases between 2 SNPs sharing > 99.9% sequence identity with 20154871 were measured.

## Results

### Distributions of IPD serotypes, pilus determinants, and resistance features

**Figure 1:**
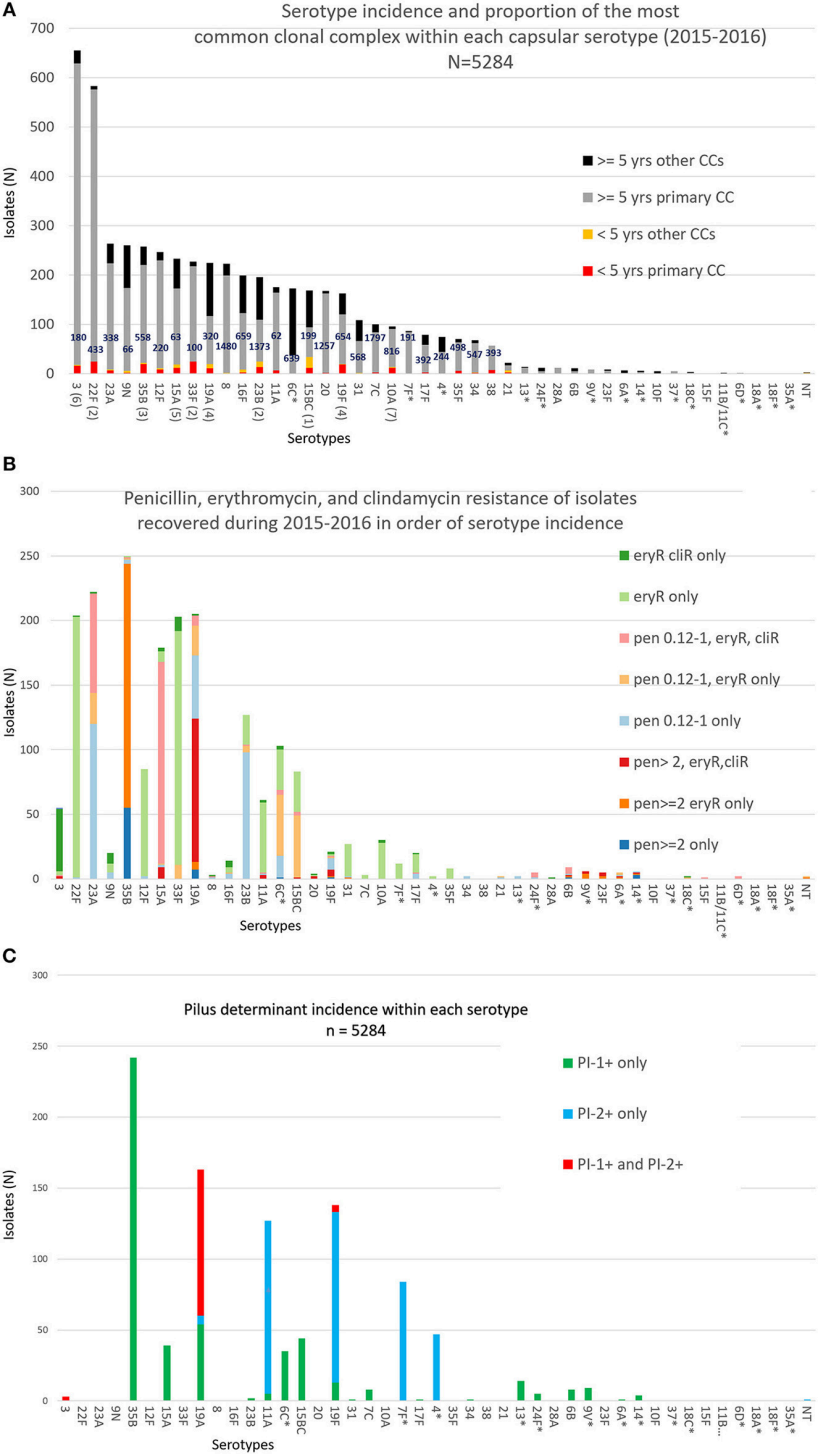
**(A)** Serotype frequency, patient age distributions, and proportion of most common clonal complex (CC) within each serotype for 5284 isolates. The ranking of the 10 serotypes causing the most IPD in individuals <5 yrs of age is shown in parentheses. The major MLST-based clonal complex within each serotype is shown within each column. There were no pediatric isolates within serotypes indicated with an asterisk. **(B)** Numbers of isolates within each serotype with individual combinations of penicillin, erythromycin, and clindamycin resistance phenotypes/ **(C)** Pilus backbone determinant frequency (presence or absence of PI-1 and/or PI-2) within each serotype.

There were 44 serotypes detected among the 5284 case isolates for which we had patient ages (Figure 1A), with 27 serotypes accounting for 22 or more isolates (ranging from 22 isolates within serotype 21 to 657 serotype 3 isolates). PCV13 serotypes 3, 19A, and 19F accounted for 20.4% (1089/5334) of the isolates from all ages (age information was lacking for 51 isolates) and 19% (58/305) of the isolates from children <5 yrs. No isolates of PCV13 serotypes 6C, 7F, 4, 9V, 6A, 14, and 18C from children <5 yrs were recovered (Figure 1A). There were no isolates of PCV13 serotypes 1 and 5 among the 5334 isolates.

For 19 of the 28 serotypes found in children <5 yrs, the most abundant (primary) clonal complex was also predominant for the remaining isolates from all ages (includes serotypes 3, 22F, 23A, 35B, 12F, 15A, 33F, 19A, 23B, 11A, 19F, 7C, 10A, 17F, 35F, 34, 38, 28A, and 23F; there was only a single isolate from <5 yrs for each of the serotypes 28A, 6B, and 23F) (Figure 1A). Except for the genetically diverse serotypes 6C and 19A, each serotype accounting for > 22 isolates contained a single major broad clonal complex that accounted for the majority of isolates.

**Figure 2:**
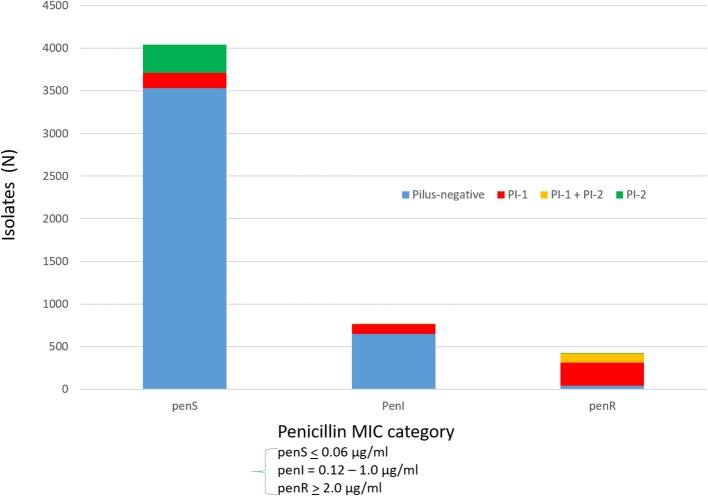
Association of PI-1 and PI-2 (positive for backbone pilus protein genes *rrgA* and *pitB*, respectively) with penicillin MICs (penS ≤ 0.06 μg/ml, penI = 0.12–1 μg/ml, penR ≥ 2 μg/ml).

Serotypes had widely varying proportions of different combined resistance phenotypes to penicillin, erythromycin, and clindamycin (includes both constitutive and inducible resistance), primarily inferred by the presence of specific PBP types, *mef*, and *ermB*. For example, serotype 3 accounted for most isolates with the relatively uncommon erythromycin-resistant (eryR), clindamycin-resistant (cliR), penicillin-susceptible (penS) phenotype, while 22F, 12F, and 33F comprised the majority of the eryR, penS phenotype (Figure 1B). Pilus determinant distributions were not highly correlated to serotype frequency (Figure 1C), however positivity for the PI-1 pilus backbone protein determinant *rrg* was highly associated with penicillin-nonsusceptibility (Figure 2) as previously observed (Sjöström et al., 2007). Of the 4040 penS isolates, only 184 (4.6%) were *rrg—*positive (PI-1+). In contrast, 120/768 (15.6%) of the penI and 379/425 (89.2%) of the penR isolates were PI-1+ with the addition of isolates that were both PI-1+ and PI-2+ (*p* < 0.0001).

**Figure 3:**
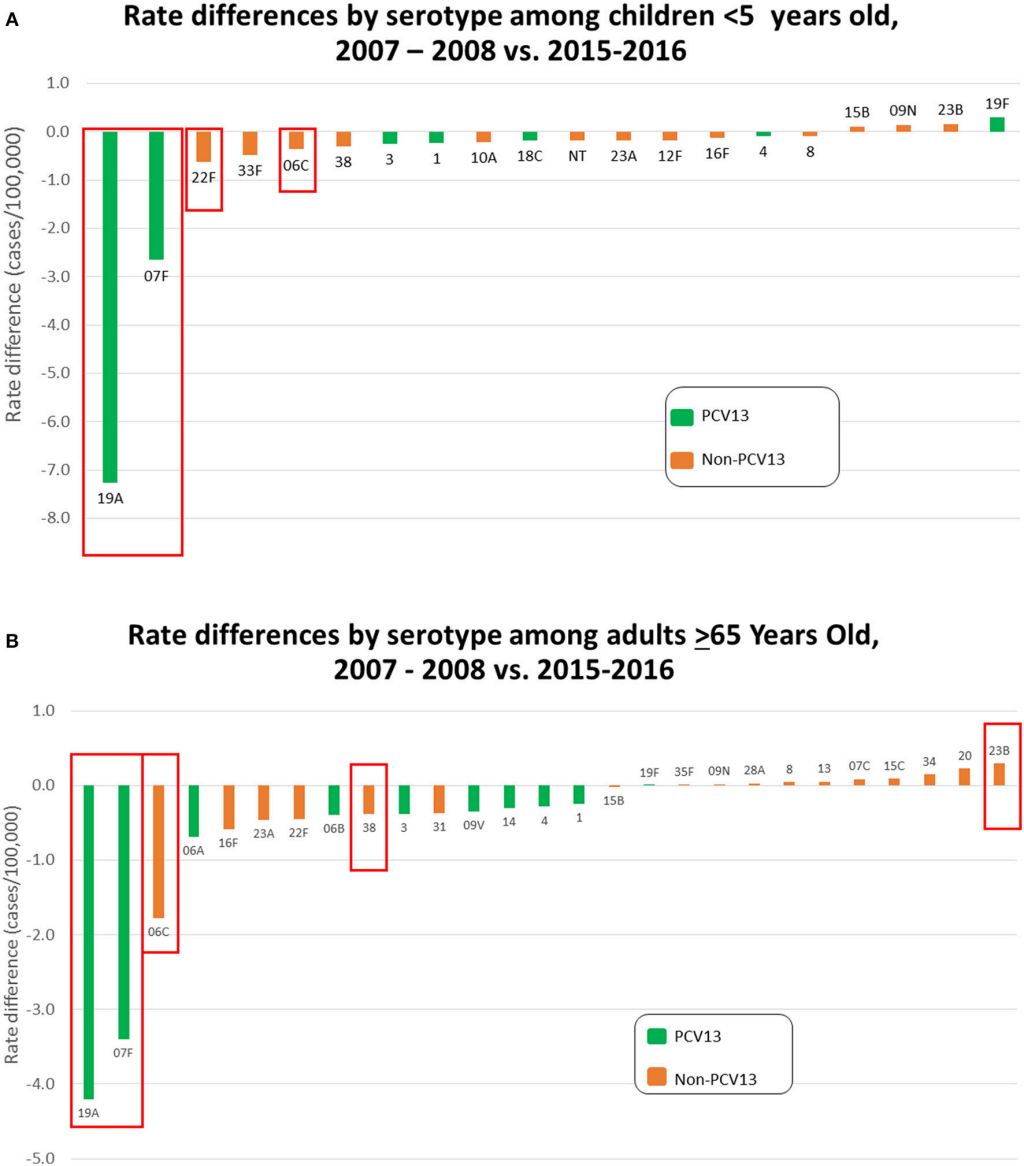
**(A)** Rate differences by serotype among children < 5 yrs old comparing July 2007–June 2009 vs. July 2015–June 2016. Serotype 6C is grouped with the PCV13 serotypes. **(B)** Rate differences by serotype among adults > 65 yrs old comparing 2007–2008 vs. July 2015 – June 2016. Serotype 6C is grouped with the PCV13 serotypes.

Figures 3A,B depict rate differences by serotype comparing IPD incidence pre-PCV13 (2007-2008) to post-PCV13 (2015-2016) in children < 5 yrs and in adults >65 yrs. PCV13 serotypes, with the exception of serotype 19F, decreased during 2015–2016 relative to 2007–2008 in young children and elderly adults. Significant reductions were observed for serotypes 19A and 7F, which were the predominant IPD serotypes during 2007–2009, and for serotype 6C. Slight increase in non-PCV13 serotypes and PCV13 serotype 19F were observed among both young children and elderly adults in 2015–2016 when compared to 2007–2008 with a significant increases for serotype 23B among the elderly. Serotype-specific incidence changes showed no consistent correlations with antimicrobial resistance or the presence/absence of pili.

**Figure 4:**
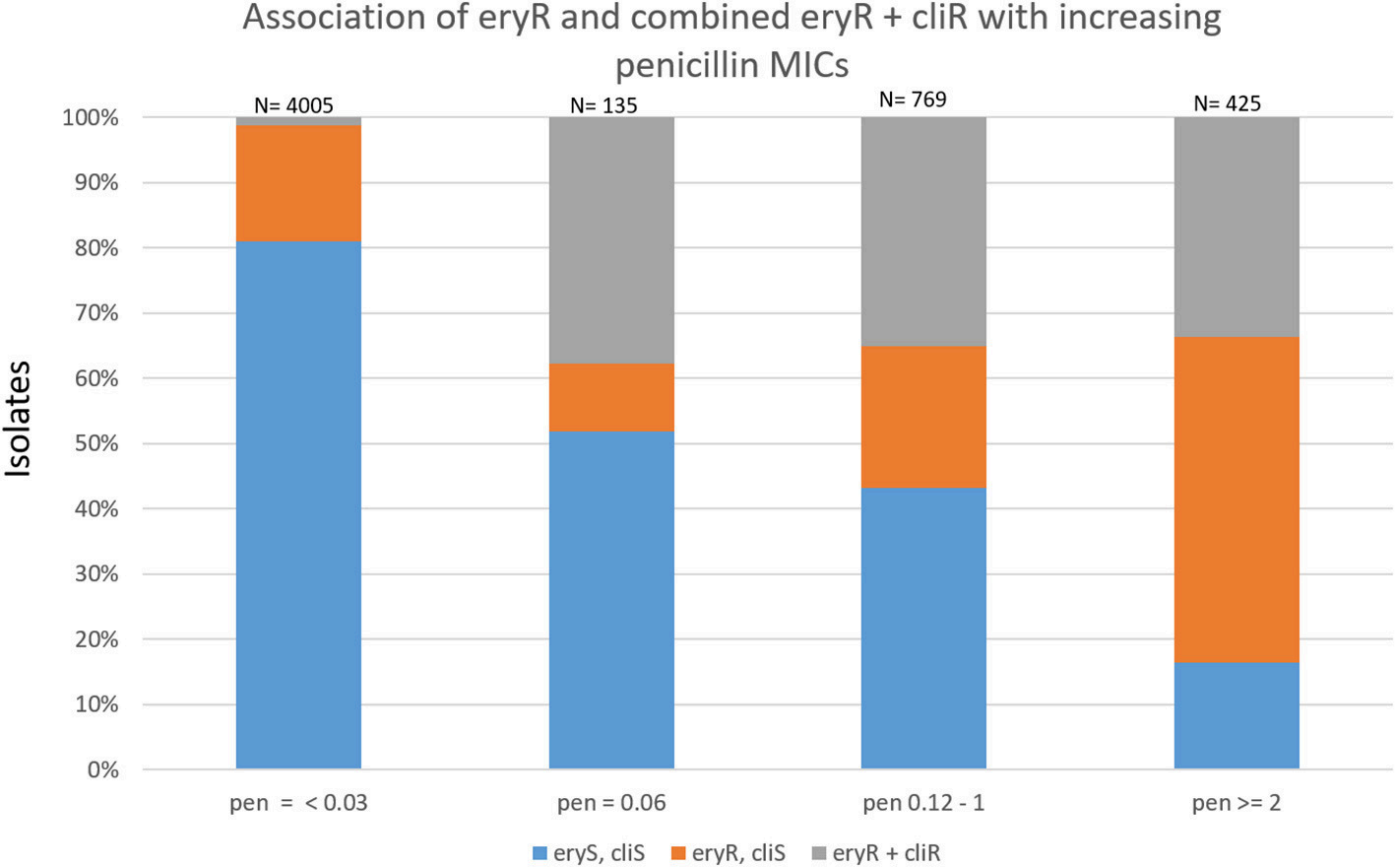
Association of resistance to erythromycin and clindamycin with increasing penicillin MICs within 5334 ABCs isolates recovered during 2015–2016.

**Figure 5:**
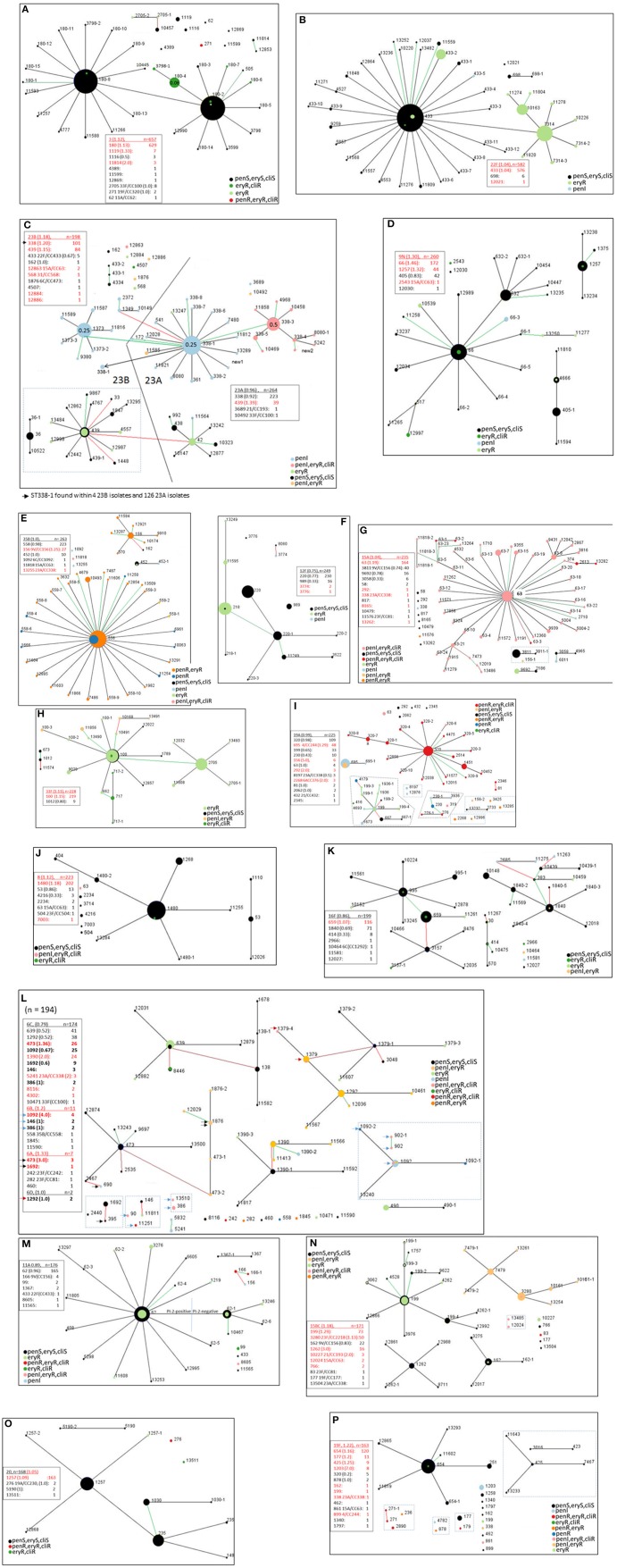
**(A–P)** Ten locus MLST eBURST of the 17 predominant serotypes (15B and 15C included together; serotypes 6A, 6B, and 6D included with serotype 6C). For each serotype, a descriptive section is provided in the text. Designations with dashes simply indicate differences in 1–3 of the PBP loci (e.g., ST180-2 vs. ST180-8, ST433 vs. ST433-1) Each lists a legend where the serotype is underlined and indicates the ratio of year 2016 isolates of that serotype to 2015 isolates in parentheses. Below each serotype individual clonal complexes are listed, also with the ratio of 2016 to 2015 isolates. Entries where there are more year 2016 isolates than year 2015 isolates are indicated in red font. Strain complexes that have only been detected within the post-PCV era and are likely to have originated through serotype switching are indicated, with the most likely serotype of the parental recipient strain indicated. Black, green, and red lines between nodes indicate variation at 1, 2, and 3 of the 10 loci, respectively. Clonal complexes within dotted rectangle include MLST types that differ in 1-2 of the 7 housekeeping loci, yet differ in 3 or more of the 10 locus eBURST scheme. A legend depicting relevant resistance phenotypes for penicillin, erythromycin, and clindamycin is included for each serotype. It was informative to show eBURST of serotypes 23A and 23B together **(C)**, and also between serotypes 6C, 6A, 6B, and 6D together **(L)** due to intrinsic close relationships. In 5A and 5C PBP type-driven increased penicillin MIC are depicted (see 180-4 in 5A and 338-3 in Fig 5C).

Most combined resistance to erythromycin and clindamycin was conferred by *ermB*, although 7 isolates (4 different serotypes) contained the 23S rRNA A2061G substitution conferring this resistance trait (sTable 1). The proportions of isolates with macrolide-resistance increased with increasing penicillin MICs (Figure 4). Only 19.1% of basally penicillin-susceptible isolates were macrolide resistant, while 51.1% with the “first-step” penicillin MIC (reduced susceptibility) were macrolide-resistant. Of intermediately penicillin-resistant isolates, 56.8% were macrolide-resistant, while 83.5% of isolates with penicillin MICs ≥2 μg/ml were macrolide-resistant. The distributions of combined resistance features for penicillin, erythromycin, and clindamycin are diagramatically presented within different clonal complexes for each individual serotype. Figures 5A–P depict serotypes consisting of >163 isolates, with sfigures 1–17 depicting the remainder. For several serotypes whole genomic based phylogenies are depicted (sfigures 18–25).

In several instances, differences within PBP type (which provide three determinants in the 10 locus eBURST scheme) coincided with additional resistance features besides beta lactam resistance (shown for macrolide and clindamycin-resistance). For example, this is evident within serotypes 3 (ST180-4 in Figure 5A), 22F (ST433-2 in Figure 5B), 23A (ST338-3 in Figure 5C), and 6C (ST1390 in Figure 5L).

The highest penicillin MICs (>4–8 μg/ml, exact MICs distinguishing between MICs of 0.12–8.0 μg/ml only provided in sTable 1), combined with *ermB/mef* –conferred resistances, were restricted to a related (CC320) set of 116 serotype 19A, 19F, and 3 (only 7 total isolates of serotypes 3 and 19F) strains (Figures 1B, 5A,I,P) that were positive for both of the two pilus protein gene queries (Figure 1C) as well as both major macrolide-resistance determinants (*mef* and *ermB*) (sTable 1). Serotype 35B accounted for the most penicillin resistance (244 isolates with 189 additionally eryR). The penR 35B strains and the remaining penicillin-nonsusceptible isolates (MICs > 0.12 μg/ml) were primarily comprised of PI-1 positive isolates with serotypes 23A, 15A, 23B, and 6C (Figures 1B,C; this association also held true for small numbers of penicillin-nonsusceptible isolates within serotypes 13, 24F, 6B and 9V). Most PI-2 positive strains were penicillin-susceptible within serotypes 11A, 19F, 7F, and 4 (Figures 1B,C).

The majority of isolates (4005/5334, 75.2%) were basally susceptible to penicillin (MIC < 0.03 μg/ml), with an additional 135 isolates that were susceptible but with reduced susceptibility (MIC = 0.06 μg/ml) (sTable 1). Such isolates usually contained “sensitive” Pbp1a and Pbp2b transpeptidase subtypes and “first step” substitutions within the Pbp2x transpeptidase region. An example of a “reduced susceptibility” PBP type included 1a2-2b0-2x111 (2-0-111) found within a clade of serotype 3/CC180 (ST180-4 and ST3798-1 indicated within Figure 5A).

Cotrimoxazole-resistance (MICs ≥ 1.0 μg/ml) was found in 1005 of the 5334 isolates (18.8%) due to mutations within *folA* and/or *folP* (Metcalf et al., 2016a) and was also more commonly associated with penicillin-nonsusceptible isolates (49.1% of those with penicillin MIC ≥ 0.12 vs. 10.1% in penicillin-susceptible). Of 5334 isolates, 632 (11.8%) were positive for tetracycline-resistance genes. Of these, 475 (75.2%) had penicillin MICs ≥ 0.12 μg/ml. There were 6 levofloxacin-resistant isolates (sTable 1), all containing substitutions within both ParC and GyrA (3 unrelated isolates of ParC:S79F+GyrA:S81F, 1 ParC:S79F+GyrA:E85G, 1 ParC:D83N+GyrAS81F, 1 ParC:D83Y+GyrA:S81F).

### Overview of individual serotypes and serotype switch events

The sections that follow provide general depictions of clonal complexes, resistance features, and recently documented (2002 or later) putative serotype-switch variants within each serotype. sTable 2 provides a listing of year 2015–2016 switch variant progeny, along with their major MLSTs, PBP types, and resistance features. Each CC/serotype combination is designated as a category of switch variant.

We quantitated strain complexes only detected in the post-PCV era (2002 or later) that potentially arose through serotype-switching events, as documented within the global MLST database and in our own surveillance records. We identified 57 categories that were defined by serotype/CC combinations (e.g., 23B/CC338, 6C/CC338 etc.) with no documented record prior to 2002 and they accounted for 404 (7.7%) of the 5334 isolates (sTable 2). Twenty-two of the 57 categories, accounting for 174 isolates, could be described as “vaccine-escape”, where PCV13 serotypes (including 6C) were replaced with a non-PCV serotype. Seven of the 57 categories of progeny (23B/CC338, 15BC/CC3280, 19A/CC244, 15A/CC156, 4/CC439, 35B/CC156, and 15BC/CC156) accounted for 325 (80.4%) of the putative serotype-switch progeny (sTable 2). Each of the progeny strains are depicted within relevant eBURST diagrams included in Figures 5A–P and sFigures 1–17.

Certain strain complexes that were represented by numerous progeny accounted for multiple switch events. For example, examination of PBP and MLST alleles within 35B/CC156 progeny revealed that this complex originated from at least 3 independent serotype-switch events (Chochua et al., 2017). Similarly, the CC338 strains 23B/ST1373 and 23B/ST338 originated from different ancestral events.

Seventeen of the categories accounted for 101 progeny isolates of PCV13 serotypes 19A (52 isolates), 4 (28 isolates), 3 (11 isolates), 6C (4 isolates), 19F (3 isolates), 6A (2 isolates), and 6B (1 isolate). The number of PCV13 serotype switch progeny is inflated substantially by the number of 19A/ST695 (CC244) isolates (Figure 4I), even though overall 19A incidence (Figures 3A,B) and its predominant 19A/CCs (CCs320, 695, and 199) have continued to declinex (Kim et al., 2016; Metcalf et al., 2016b).

The most promiscuous serotype recipient CCs, accounting for 6-7 different putative progeny serotypes, were CCs 156 (7 serotypes), 63 (7 serotypes), and 338 (6 serotypes). CC338 accounted for the most putative progeny, primarily through the rapid post-PCV emergence of 23B/CC338 (23B/ST1373) (Andam et al., 2017).

### 35B/CC558 as a substrate for recombination

There were numerous examples of apparent hybrid strains that exhibited typing pipeline features highly associated with two different strains of known lineages (data intuited from sTable 1). Such strains included many putative serotype-switch strains within sTable 2, but also included other strains with altered beta lactam MICs conferred by horizontal transfer of PBP loci. Several recombinant strains that involved the 35B/CC558 lineage were easily recognized due to PBP typing determinants and MLST loci that are highly specific for this lineage (Table 1). The MLST determinants *recP44, xpt77*, and *ddl97* were all initially documented from within invasive 35B/ST558 circulating within the United States during 1995–2001 (Beall et al., 2002). Similarly, we have found that the 3 resistance-conferring mosaic PBP genes associated with PBP type 4-7-7 are highly associated with 35B/CC558, although as indicated within Table 1, 2b-7 is also found within year 2015–2016 serogroup 6/CC1092 and 19A/CC230 IPD isolates from 1998-present. Table 1 depicts 16 putative recombination events (multifragment recombinations counted as single event) that implicate 35B/CC558 as a genetic donor (of *cps35B*, MLST loci, or PBP type determinants) in 15 genetic exchanges and as a genetic recipient (of *cps6B*) in a single exchange, resulting in the indicated progeny strains shown in the right column. Each exchange is defined by 1–3 specific double crossover events deduced in 1–25 progeny isolates.

**Table 1:** Recombination between pairs of strains that include 35B/ST558^A^.

**Strain**	***recP*^B^**	***xpt*^C^**	**2B^D^**	***ddl*^E^**	***gdh***	***aroE***	***gki***	***spi***	**1A^F^**	***cps***	**2X^G^**	**Cotrimoxazole-MIC determinants**	**Pilus1**	**accessory resistance deteminants**	**Progeny strain, n, (progeny isolate(s) ID examined)**
35B/ST558	44	*xpt77*	**2B-7**	***ddl97***	*gdh12*	*aroE18*	*gki4*	*spi14*	**1A-4**	***cps35B***	**2X-7**	FolAwt,FolPwt	+	−	Not applicable
						+								
1.9V/ST156 = (recipient)	1	*xpt8*	2B-12	*ddl1*	*gdh11*	*aroE7*	*gki10*	*spi6*	**1A-4 33,509**	***cps35B*** **33,509**	2X-7 4,896	FolA100L; FolPins178	+	*mef*	= **35B/ST156**, ***n*** = **25**^I^ **(20154871)**
2.9V/ST156 = (recipient)	1	*xpt8*	**2B-7 6,626**	**97 6,626**	*gdh11*	*aroE7*	*gki10*	*spi6*	**1A-4 32,405**	**cps35B 32,405**	2X-18	FolA100L;FolPins178	+	*mef*	= **35B/ST10174**, ***n*** = **1 (20170747)**
3.23A/ST338-1 (recipient)	**44 35,060**	*xpt6*	2B-1	*ddl8*	***gdh12*** **11,439**	*aroE7*	*gki8*	*spi1*	1A-0	***cps35B*** **23,270**	2X-1	FolAwt;FolPins195	−	−	= **35B/ST13255**, ***n*** = **1 (20172864)**
4.6C/ST1092 = (recipient)	1	*xpt19*	7 ^negative^	*ddl14*	*gdh13*	*aroE2*	*gki2*	*spi6*	1A-6	***cps35B*** **28,071**	**2X-36**^H^ **13,889**	FolA81H,83I,94D, 100L;FolPins195	+	−	= **35B/ST1092**, ***n*** = **1 (20160919)**
5.15A/ST11818 = (recipient)	12	*xpt21*	2B-31	*ddl20*	*gdh5*	*aroE2*	*gki36*	*spi17*	**1A-4 28,076**	***cps35B*** **28,076**	114	FolAwt;FolPins195	−	*ermB/tetM*	= **35B/ST11818**, ***n*** = **1 (20161647)**
6.15A/ST63 = (recipient)	12	*xpt21*	**2B-7 3,125**	*ddl14*	*gdh5*	*aroE2*	*gki36*	*spi17*	**1A-4 13,185**	*cps15A*	**2X-7 16,588**	FolAwt;FolPwt	−	*ermB/tetM*	= **15A/ST63penR1**, ***n*** = **1 (201554582)**
7.15A/ST63 = (recipient)	12	*xpt21*	2B-74	*ddl14*	*gdh5*	*aroE2*	*gki36*	*spi17*	**1A-4 17,503**	*cps15A*	**2X-7 22,357**	FolAwt;FolPins178	−	*ermB/tetM*	= **15A/ST63penR2**, ***n*** = **1 (20152153)**
8.15A/ST63 = (recipient)	12	*xpt21*	2B-27	*ddl14*	*gdh5*	*aroE2*	*gki36*	*spi17*	1A-146	*cps15A*	**2X-7 1,424**	FolAwt;FolPins178	−	*ermB/tetM*	= **15A/ST63penR3**, ***n*** = **1 (20166571)**
9.15A/ST7473 = (recipient)	12	*xpt21*	**2B-7 14,961**	**97 14,961**	*gdh7*	*aroE2*	*gki36*	*spi17*	1A-13	*cps15A*	2X-73	FolA81H,100L; FolPins178	−	*ermB/tetM*	= **15A/ST13486penR4**, ***n*** = **1 (20170475)**
10.15A/ST63 = (recipient)	12	21	2B-7 565	14	5	2	36	17	24	15A	114	FolAwt;FolPwt	−	*ermB/tetM*	= **15A/ST63**, ***n*** = **1 (20164351)**
	12	21	2B-7 565	14	5	2	36	17	148	15A	138	FolAwt;FolPins195	−	*ermB/tetM*	= **15A/ST63**, ***n*** = **1 (20170474)**
	35	21	2B-7 565	14	5	2	36	17	148	15A	2	FolAwt;FolPwt	−	*ermB/tetM*	= **15A/ST13486**, ***n*** = **1 (20173651)**
11.15A/ST63 = (recipient)	**44 4,665**	*xpt21*	2B-27	*ddl14*	*gdh5*	*aroE2*	*gki36*	*spi17*	IA-24	*cps15A*	2X-13	FolAwt;FolPins178	−	*ermB*/*tetM*	= **15A/ST12867**, ***n*** = **2 (20164836 and 20171132)**
12.33F/ST10491 = (recipient)	12	*xpt3*	2B-23	*ddl18*	*gdh12*	*aroE5*	*gki29*	**14** >**26001**	**4** >**24,618**	*cps33F*	**2X-7 15,031**	FolAwt;FolPins189	−	*mef*	= **33F/ST10168penNS**, ***n*** = **2 (20171854 and 20163481)**
	12	*xpt3*	2B-23	*ddl1*	*gdh12*	*aroE5*	*gki29*	**14** >**26001**	**4** >**24,618**	*cps33F*	**2X-7 15,031**	FolAwt;FolPins189	−	*Mef*	= **33F/ST13491penNS**, ***n*** = **1 (20171636)**
13.23A/ST338-3 = (recipient)	6	***xpt77*** **7,886**	2B-1	*ddl8*	*gdh13*	*aroE7*	*gki8*	*spi1*	1A-19	*cps23A*	2X-24	FolAwt/FolPwt	−	*ermB/tetM*	= **23A/ST11858**, ***n*** = **1 (20164311)**
14.6C/ST639 = (recipient)	*6*	***xpt77*** **16,844**	2B-0	*14*	*5*	*7*	*8*	*6*	2	*cps6C*	0	FolA81H,100L; FolPins178	−	−	= **6C/ST12879**, ***n*** = **1 (20165616)**
15.6B/? = (donor)	44	*xpt77*	2B-7	*ddl97*	*gdh12*	*aroE18*	*gki4*	*spi14*	1A-4	*cps6B* 13,743	2X-7	FolA81H,100L; FolPins178	+	−	= **6B/ST558**, ***n*** = **1 (20155436)**
**Table 1B Recombination between 35B/452 (cps35B donor) and 9V/ST162 (recipient)**.															
35B/ST452^J^	*recP1*	*xpt48*	2B-0	*ddl14*	*gdh9*	*aroE7*	*gki19*	*spi14*	1A-0	*cps35B*	2X-0	FolAwt;FolPwt			
						+									
9V/ST162^J^	*recP1*	*xpt8*	2B-0	*ddl14*	*gdh11*	*aroE7*	*gki10*	*spi14*	1A-0	*cps35B*	2X-0	FolAwt;FolPwt	*rrg*		= **35B/ST162**, ***n*** = **1, (20172564)**

A *Sizes of recombinational fragments found in progeny sharing 99.9% - complete identity with the indicated 35B/ST558 donor reference strain are indicated below red or green entries. All other markers are presumed to originate within the parental strain (left column). Adjacent green entries indicate markers co-transferred on a single fragment (or genetically linked within 35B/ST558). Italicized entries indicate chromosomal gene fragments for MLST (recP, xpt, ddl, gdh, aroE, gki, spi) or capsular biosythetic loci*.

B *recP44 was observed among 223 of the 5335 isolates. All of these were 35B/CC558 strains except for the 4 indicated recombinant isolates (strains 15A/ST12867, 35B/ST13255, and 6B/ST558)*.

C *xpt77 was observed among 224 of the 5335 isolates. All of these were 35B/CC558 isolates except for the 3 indicated recombinants (strains 23A/ST11858, 6B/ST558, and 6C/ST112879). The xpt77– containing chromosomal fragment sizes (in base pairs) in 2 progeny strains are indicated below*.

D *The PBP type marker 2B-7 was observed within 239 of the 5335 isolates. Eight of these are suspected recombinants shown above. Also there were 15 6C/ST1092, 2 6B/ST1092, and 5 19A/CC230 isolates. The remaining 211 isolates were 35B/CC558 isolates. The 2 green entries each indicate that the 2B-7 and ddl97 determinants were co-transferred into the recipient on the same chromosomal fragment. In previous ABCs isolates dating back to 1998, 2B-7 has been solely observed among 35B/CC558, serogroup 6 CC1092, and 19A/CC230 isolates. In entry 4 the 835 bp 2B-7 encoding region shared identity with various serogroup 6/CC1092 isolates, but only 98.8% identity with the 35B/ST558 reference strain. Three 15A/CC63 isolates revealed the same pbp2b allele, with a potential 565 bp internal fragment originating from a 35B/CC558 donor*.

E *ddl97 was found in 218 of the 5335 isolates. All of these were 35B/CC558 isolates except for the 3 recombinant isolates shown (35B/ST10174, 15A/ST13486penR, and 6B/ST558). The 2 green 2B-7 entries indicate co-tranferred 2B-7 and ddl97 determinants. The size of the fragment originating from 35B/CC558 resulting in the fusion 2b-74 subtype is also indicated*.

F *The PBP type marker 1A-4 was observed among 249 of the 5335 isolates. Of these 216 were 35B/CC558. The remaining 33 are depicted in this table, including 26 35B/CC156 isolates derived from a 9V/ST156 recipient, a 35B/ST11818 derived from a 15A/ST11818 recipient, 2 penR 15A/ST63 strains, 2 33F recombinants, and the 6B/St558 strain*.

G *The PBP type marker 2x-7 was associated with 35B/CC558 with the exception of recombinant strains listed within this table*.

H *Although still resulting in a change from 2x-7 to 2x-36, the full length pbp2x gene in the 35B/ST1092recombinant differed by a single base from the 1762 bp pbp2x gene from the donor 35B/ST558 reference and shared only 95.9% sequence identity with the putative 6C/ST1092 recipient reference sequence. This change in PBP subtype was associated with a second double crossover event closely linked with the 6C to 35B cps locus switch*.

I *These were 20 35B/ST156 and 5 were 35B SLVs of ST156 that shared same recombination pattern*.

J *The left hand column indicates the two strains (red font 35B and black font 2nd strain) with 35B/ST162 indicated as recombinant progeny strain*.

There were 5 clear instances of serotype switching events involving a 35B/CC558 parental donor strain, involving 5 different donor serotype lineages. The most important serotype 35B switch variant, accounting for 25 isolates, arose from a single event where the 9V/ST156 *pbp2x-cps9V-pbp1a* region was replaced with the corresponding sequences from 35B/ST558 through two closely situated recombination events (Table 1, number 1). This strain was initially reported from a year 2012 ABCs isolate (Metcalf et al., 2016b) and was subsequently found within all 10 ABCs surveillance sites during 2015–2016 (Chochua et al., 2017). The second serotype switch, also determined previously to share the same 2 parental lineages (Metcalf et al., 2016b), resulted in a 35B/ST10174 strain that was discovered as a year 2009 IPD isolate. Within this strain two unlinked recombination events were revealed; one encompassing the contiguous 1A-4 and *cps35* loci, and the other encompassing the contiguous 2B-7 and *ddl97* markers as an example of *ddl* “hitchhiking” with a resistance-conferring *pbp2b* allele (Enright and Spratt, 1999). We have subsequently recovered two 35B/ST10174 IPD isolates recovered during 2016-2017. The third serotype-switch shown in Table 1 implicate a 35B/CC558 *cps35B* donor on the basis of 3 unlinked recombination events that replaced the *recP, gdh*, and *cps* regions of the common 23/ST338/PBP type 0-1-1 with *recP44, gdh12*, and *cps35B* resulting in a 35B/ST13255/PBP type 0-1-1 progeny strain. Table 1, number 4 involved a 35B/CC558 *cps35B* donor and a serogroup 6 recipient where one recombination event replaced *cps6* and the second closely linked double crossover resulted in a hybrid *pbp2x* fusion gene creating a new PBP2x subtype (2x-36). The fifth serotype switch event involved a 35B/CC558 *cps35B* donor and a 15A/ST11818 recipient. It is interesting that there were 4 15A/ST11818 isolates recovered, 3 of which shared the common 15A/CC63 PBP type 24-31-114. The PBP type of the progeny 35B/ST11818 (4-31-114) and its *cps35B* region sequence are consistent with a gene replacement event effecting a serotype switch event and a change in PBP type. Here, a single chromosomal fragment carrying *cps35B* and the 3′ 544 codons of *pbp1a* (encompassing PBP subtype 1a-4) from a 35B/CC558 strain replaced the corresponding region from a 15A/ST11818/PBP type 24-31-114 strain.

A sixth serotype switch depicted in Table 1 (entry 17) involved an unknown lineage *cps6B* locus donor and a 35B/ST558 recipient strain (last row). Comparison of the *cps* locus from this recombinant to a *cps6B* reference locus (CR931639) and the 35B/ST558 *cps35B* reference revealed potential crossover sites corresponding to bases 2321 (within *wzg*) and 16033 (immediately downstream of 4 gene rhamnose biosynthetic gene cluster) of the *cps6B* reference, with 99.2% sequence identity shared within the 13,743 bp overlap of these two *cps6B* loci. As expected, sequences upstream and downstream of the *cps6B* locus that encompassed the full-length *pbp2x* and *pbp1a* genes in the recombinant strain were highly similar to the 35B/ST558 recipient reference strain (>99.5 % sequence identity over 14,433 bp flanking the *cps6B* switch fragment, including complete identity with full-length 2160 bp *pbp1a* and 1762 bp *pbp2x* genes).

The remaining 9 entries in Table 1 (entries 6-14) do not involve *cps* switches, but introduced changes in MLST and/or PBP loci that were observed in the progeny. Except for entry 10, where the short length (565 bp) of the *pbp2b* putative replacement fragment does not provide strong association with a 35B/CC558 donor, these putative recombination events appeared non-subjective, involving replacement fragments of 1424 bp - > 26,001 bp that closely matched the 35B/ST558 reference. Four of these hybrid 15A/CC63 strains (entries 6-9) had higher predicted MICs for beta lactams due to the introduced changes in PBP type. Replacement of 2- 3 PBP markers resulted in a change from the median 15A/CC63 pen MIC from 0.25 to 2 μg/ml (entries 6 and 7). The predicted change from PBP type 13-115-73 (the closest matching PBP type among our 15A isolates) to 13-7-73 results in change of pen MIC from 1 to 2 μg/ml. All 3 33F hybrid progeny appeared to result from an identical strain mixing that effected 3 large and unlinked gene replacements within a 33F/ST10491 recipient (entry 12 in Table 1). Two of these replacements dramatically increased beta lactam MICs through changing the 33F/ST10491 PBP type from 2-23-6 (associated with pen MIC < 0.03 μg/ml) to 4-23-7 (pen MIC = 1 μg/ml).

Another independent serotype 35B switch variant described in Table 1B (isolate 20172564, 35B/ST162/PBP type 0-0-0) potentially originated from a switch event involving the penS 9V/ST162 (PBP type 1-0-0) recipient and a 35B/ST452 (PBP type 0-0-0) donor (Chochua et al., 2017).

### Genotypic features of interest within individual serotypes

#### Serotype 3

Despite being a PCV13 serotype, serotype 3 was the major IPD serotype overall during 2015–2016. It is interesting that the clade centering upon ST180-2 coincides with the recently described globally emergent CC180 clades I-β and II (Azarian et al., In Press). STs 180-4 and 3798-1, which feature *ermB* expression combined with reduced penicillin-susceptibility due to a first step PBP-2x substitution (0.06 μg/ml indicated), coincides with the finer-detailed phylogeny shown sFigure 18.

Three serotype switch variant lineages (11 isolates) were putatively derived from 33F/ST2705, 19F/ST271, and 11A/ST62 recipient strains (sTable 2, categories 19, 44, 48). The 2 type 3/ST271 strains, isolated during 2015 and 2016, shared the same MLST and resistance features (penR, eryR, cliR, cotR, tetR) as the two type 19F/ST271 isolates in this study (sTable 1). These 2 serotype 3 isolates differed by only 10 SNPs, shared identical *cps3* operons, PBP type 17-16-47, and flanking regions, indicating that the 2 isolates arose from the same serotype switch event. The closest matching PBP type (13-16-47) that we have found within ABCs isolates was from 2 19F/ST271 isolates recovered during 2004 and 2012 (data not shown).

#### Serotype 22F

Serotype 22F displayed 2 significant *mef* + lineages (196 isolates) that included the ST7314-focused clade and ST433-2 (Figure 5B). These eBURST-based clades are in agreement with finer phylogenetic resolution and generally show much deeper branching within the ST433 clade relative to the ST7314 clade which is consistent with more recent emergence of the major macrolide-resistant ST7314 clade (sFigure 19).

#### Serotypes 23A and 23B

Serotypes 23A and 23B are depicted together (Figure 5C) since two broad MLST-based clonal complexes (CC338 and CC439) represent the majority of both serotypes (99.2% of 23A and 93.4% of 23B). Although ST338 was first noted in association with serotype 23F, within ABCs it has been found almost exclusively within 23A even before PCV7 implementation (Pai et al., 2005a). This observation is potentially indicative of 2 shared major ancestral lineages encompassing both serotypes. Clonal shifts occurred within 23A, 23B and other non-PCV serotypes during the post-PCV7 years such that more ABCs isolates were of the penicillin-nonsusceptible CC338 (Gertz et al., 2010).

Although, 23A and 23B ranked 3rd and 12th in overall incidence, respectively, there were 25 23B isolates from children and only 9 23A isolates from children. Serotype 23B was the only NVT serotype that increased in children (also in the elderly).

It is interesting that of the numerous STs represented in the broad complexes CC338 and CC439, one (ST338-1) was found in both serotypes (there were 4 23B/ST338-1 isolates and 126 23A/ST338-1 isolates). Phylogenetic analysis was consistent with 23B/ST338-1 arising from at least one serotype switch with a 23A/ST338-1 recipient (sFigure 20, sTable 2, category 1), however the major CC338 genotype within 23B was ST1373, which has only been traced within ABCs 23B isolates in the post-PCV7 era (Gertz et al., 2010; Metcalf et al., 2016b) and was originally reported from a 19F isolate recovered in 2001 (www.mlst.net). It is interesting that 23B/ST1373 has been associated with a divergent *cps* locus (Andam et al., 2017) previously described as sequence subtype 23B1 (Kapatai et al., 2016). The short branch lengths among the majority of the 23B/ST1373 isolates are consistent with the recent emergence and expansion of this strain complex (sFigure 20).

Within serotype 23A there were 2 separate clades originating within ST338-1 and ST338-3 that differ between 2 of 3 PBP loci (0-1-1 vs. 19-1-24), which corresponds to increased antibiotic resistance in the ST338-3 focused clade (higher penicillin MIC of 0.5 μg/ml and *ermB-*conferred resistance to erythromycin and clindamycin) (Figure 5C).

There was one penicillin-nonsusceptible (PBP type 0-0-1) variant (ST10492) of the major 33F/CC100 lineage within serotype 23A (sTable 2, category 21). Within serotype 23B there were 9 putative switch variants representing major CCs of serotypes 22F (5 isolates), 15A (2 isolates), 31 (1 isolate), and 6C (1 isolate) (sTable 2, categories 33, 32, 43, 55).

#### Serotype 9N

One PBP variant (ST66-3) was comprised of 5 penicillin-nonsusceptible isolates. One of the 7 *ermB*+ isolates was a SLV (ST2543) of the major 15A/ST63 (sTable 2, category 28).

#### Serotype 35B

The serotype 35B isolates include the recently described 35B serologic subtype 35D caused by mutations within the *wciG35B*-encoded acetyltransferase gene (Chochua et al., 2017; Geno et al., 2017). Potential 35D isolates represented 7.7% of the serotype 35B isolates based solely upon substitutions and indels within *wciG35B*. Indels accounted for 2.3% of these isolates and were generally reliably distinguished as serotype 35D. The variety of individual *wciG35B* changes (not shown), almost all of which occurred within single isolates, possibly indicates the lack of a selective advantage for this serologic variant of 35B.

The proportion of penicillin-nonsusceptible 35B ABCs isolates has steadily increased throughout most of the PCV era (Gertz et al., 2010; Chochua et al., 2017) primarily due to continued expansion of 35B/CC558. Most (220/260, 85%) 35B isolates were of penicillin-resistant CC558 (Figure 5E) that was first described among pre-PCV7 IPD isolates (Beall et al., 2002), with 165 of the 220 isolates also harboring *mef*.

CC156/35B included isolates from at least 3 independent serotype switch events, including the previously described 35B/ST156 major variant and the independent resistant switch variant 35B/ST10174 carrying the closely linked *ddl97* and *pbp2b-*7 markers (Enright and Spratt, 1999) derived from the 35B/ST558 serotype donor strain (Metcalf et al., 2016b; Chochua et al., 2017). As with ST10174, ST162 shares 6 of 7 housekeeping loci with ST156, however 35B/ST162 was likely to have originated from a third serotype switch between penicillin-susceptible 35B and 9V strains (sTable 2, category 14, discussed more fully in below section).

Three additional serotype-switch 35B isolates were evident, involving putative recipient 6C/CC1092, 15A/CC63, and 23A/CC338 strains (sTable 2, categories 3, 31, 46). The 21 pediatric isolates were comprised of CC558 (19 isolates) and the previously described major serotype switch ST156 variant (2 isolates with PBP type 4-7-12) (Metcalf et al., 2016b; Chochua et al., 2017, Table 1).

#### Serotype 12F

CC220 was also the major CC of 12F in ABCs prior to PCV7 (Gertz et al., 2003), with most CC220 isolates either macrolide-susceptible (ST220) or *mef*+ (ST218) (Deng et al., 2016, Figure 5F, sFigure 21). There were 16 antimicrobial-resistant isolates (to cotrimoxazole, tetracycline and chloramphenicol) of 12F/ST989 commonly found in African countries (Antonio et al., 2008).

#### Serotype 15A

Most serotype 15A isolates were CC63 (173/235 isolates, 74%), comprised of intermediately penicillin-resistant, *ermB*+ isolates (Figure 5G) that are also tetracycline-resistant and often resistant to cotrimoxazole (data not shown). The proportion of penicillin-nonsusceptible CC63 isolates within ABCs serotype 15A increased in post-PCV7 years relative to the pre-PCV years (Gertz et al., 2010). Nine CC63 isolates had a penicillin MIC of 2 μg/ml conferred by variant PBP profiles (STs 63-1, 63-2, 63-3, 2613, and 13282). Three of these profiles contain PBP types partially or completely shared with previously described penR 35B strains (ST63-1/4-7-7, ST63-2/4-74-7, and ST13486/13-7-73) (Table 1 and discussed below). Four penicillin-resistant CC63 isolates (STs 2613 and 13282) shared PBP type 34-89-147.

A recent putative serotype switch variant within CC156 was comprised of 39 antibiotic-susceptible ST3811/ST3811-1 isolates that included 4 of the 19 (21%) pediatric isolates (sTable 2, category 8). A single penI, *mef*+ ST156-1 putative switch variant was also observed (listed also as category 8 although a different switch event predicted from PBP type (15-12-13). Single switch variants were also observed of penI ST338 (major 23A MLST) and penR, *mef*+, ST11576 (SLV of the major 23F genotype ST81) (sTable 2, categories 5 and 40).

#### Serotype 33F

CC100/33F included 192 *mef*+ isolates, 11 of which were penicillin-nonsusceptible (STs 11856, 10168, 100-3, and 13491; note the tight clustering of 5 ST11856 isolates in sFigure 22). The more recent association of ST2705 with CC100 is consistent with branch lengths shown in sFigure 22. The 3 isolates (STs 10168 and 13491) with the highest penicillin MICs within 33F (1 μg/ml) shared the same PBP type 4-23-7, characterized by 2 of 3 PBP markers (1a-4 and 2x-7) originating from CC558/PBP 4-7-7 (Table 1). Eleven *ermB*+ (2 were additionally *mef*+) CC100 isolates were also observed. Six 3 locus variants (STs 673, 1012, 11574) of ST62 indicating possible common ancestry with the major 11A/CC62 were evident.

#### Serotype 19A

Although greatly decreased in incidence (Figures 3A,B) compared to pre-PCV13 years (Beall et al., 2011; Metcalf et al., 2016b) serotype 19A still ranked as the 9th most common serotype overall (tied for 4th among children) and overall was comprised of a highly antibiotic-resistant and genetically diverse isolate set (Figure 5I). Each of the 3 primary CCs (CC320, CC695, and CC199) have proportionally decreased since 2010. CC320, comprising nearly half of the 19A isolates, remained the CC associated with the highest penicillin MICs within all ABCs isolates (4–8 μg/ml), and with additional high resistances to erythromycin, clindamycin, tetracycline, and cotrimoxazole (sTable 1). Switch variant 19A/ST695 (sTable 2, category 16) was first detected in the early post-PCV7 era (Pai et al., 2005b) after which it spread throughout the U.S. (Brueggemann et al., 2007; Beall et al., 2011; Golubchik et al., 2012). CC199, the prevalent pre-PCV7 19A CC (Gertz et al., 2003), consisted primarily of *mef*+ isolates, with 3 ST4179 penR (pen MIC = 4 μg/ml) isolates. Remaining 19A CCs were previously described (Moore et al., 2008; Beall et al., 2011) including several multi-resistant 19A lineages that existed in PCV7 serotype lineages before PCV7 implementation (CCs 230, 156, 292, and 81). STs 8197 and 2268 (2 isolates each) indicate putative serotype-switch variants that potentially emerged 2002 or later (sTable 2, categories 6 and 38).

#### Serotype 8

Serotype 8 (Figure 5J) revealed one putative serotype-switch ST63 variant (penI, eryR, cliR, tetR) of the major 15A/ST63 complex (sTable 2, category 27).

#### Serotype 16F

The four outlier isolates of the three largely susceptible CCs included a penI, eryR serotype derivative (single locus variant) of a serogroup 6 lineage (sTable 2, category 57) that shared the same resistance features as 14 6C/ST1292 isolates in this study (PBP type 19-31-8, *mef*+).

#### Serogroup 6

These genetically heterogeneous isolates, consisting primarily (90%) of serotype 6C, were grouped together (Figure 5L). The arrows in Figure 5L indicate CCs representing both 6C and other serogroup 6 serotypes in this study since 6C strains appear to have emerged within several different lineages previously associated with serotypes 6A and 6B (Carvalho et al., 2009). There were 2 serotype 6D isolates (Bratcher et al., 2010), which are the first of this serotype documented in ABCs. Both of the 6D isolates were ST1379 which has been previously associated with serotype 6A (Carvalho et al., 2009). Five recent serotype-switch variants (7 isolates) were observed within serotypes 6C, 6A, and 6B (sTable 2, categories 7, 20, 39, 49, and 54]). More detailed description of the single penR 6B/ST558 variant of the major 35B complex is described below and in Table 1.

#### Serotype 11A

Two different CC62 clades were resolved through eBURST, with the major set primarily (121/131, 92%) positive for the PI2 pilus backbone protein gene (also see sFigure 23). Two serotype switch lineages were observed (sTable 2, categories 11 and 35), with 4 resistant isolates of the previously described 11A/CC156 lineage (Porat et al., 2004), and a single isolate of the major 22F/ST433 lineage.

#### Serotypes 15B and 15C

There were 171 isolates (103 of 15B and 68 of 15C) that are grouped together (Figure 5N, sFigure 24) since the two serotypes interconvert (van Selm et al., 2003). The 34 pediatric isolates (20 of serotype 15C) were distributed among 6 CCs, including 10 within the recently emerged antibiotic-resistant CC3280 (Andam et al., 2017), a single locus variant of ST2218 associated with serogroup 23 isolates recovered in Thailand during 2008 (see pubmlst.org) (sTable 2, category 15).

An additional 31 isolates were of potential recent serotype-switch CCs (sTable 2, categories 9, 22, 30, 36, and 41), including antibiotic-susceptible CC156 (22 isolates) and 15C/ST177, as well as antibiotic-resistant CC193 (*mef*+), CC63 (penI, *ermB*+*, tetM*+), and CC81 (penR, *ermB*+, cotI, *tetM*+*, cat*+).

#### Serotype 20

Two multi-resistant ST276 serotype-switch variants were observed (Figure 5O, sTable 2, category 47), one in 2015 and one in 2016. Its PBP type (17-15-18) was also observed within 4 related resistant 19A (STs 276, 230, 3936) and 19F (ST878) isolates of this study.

#### Serotype 19F

Serotype 19F is the sole PCV13 serotype that slightly increased in 2015–2016 relative to 2007–2008 within ABCs in children and the elderly (Figures 3A,B). In contrast to pre-PCV7 years, where 33% of ABCs 19F isolates were pen-NS (including 23% with MICs of 2–8 μg/ml), there were only 18 (11%) pen-NS 19F isolates recovered during 2015-2016. These penNS 19F isolates included 7 isolates (4.2%) with MICs of 2–4 μg/ml within CC320 (STs 271, 236, and 2890). The 20 pediatric isolates consisted of CC251 (18 isolates, 17 were pan-susceptible to antibiotics), ST425, and ST177 (Figure 5P). A serotype switch variant of the common serotype 4/ST899 was observed (sTable 2, category 17).

### Selected strain features within serotypes representing <110 isolates (sfigures 1–17)

Single serotype 31 switch variants of 22F/ST433 and resistant 9V/ST156 (Chochua et al., 2017) were observed (sFigure 1, sTable 2, categories 12 and 34).

Within serotype 7C there were 5 serotype-switch variant isolates (sTable 2, categories 24 and 56) that included ST177 (4 isolates) and CC1201 (1 isolate).

Serotype 10A was unusual in its over-representation of meningitis cases (unpublished data).There were 19 meningitis case isolates among the 96 isolates (19.7% compared to overall percentage of 6.3–7.1% in 2015–2016 [https://www.cdc.gov/abcs/reports-findings/surv-reports.html], which included an unusually high percentage (57.1%, 8/14) of the pediatric isolates. There was a single serotype 10A switch variant with a putative serogroup 6 recipient (sFigure 3, sTable 2, category 42).

Although under intense selective vaccine pressure in the post-PCV13 era, serotype 7F revealed no obvious serotype switch variants.

Within serotype 17F, two variants were observed that included single isolate variants potentially derived from recipient strains of 19F/ST177 and 15A/ST63 (sTable 2, categories 23 and 29).

An antibiotic-susceptible serotype 4 variant (4/ST10172) of the major serogroup 23A/23B CC439 has abruptly emerged (sFigure 6, sTable 2, category 18). This variant was first identified from infants during 2012–2013 (Metcalf et al., 2016b). The continued increase of this unusual strain 4/ST10172 within adults warrants close attention (sfigure 25).

Serotype 34 revealed three putative serotype switch variant isolates derived from genotypes of serogroup 6 and serotype 17F (ST1092 and ST392, respectively; sTable 2, categories 45 and 50).

Despite only consisting of 22 serotype 21 isolates, there were 7 pediatric isolates that included 2 meningitis case isolates. Five of the 22 isolates were from meningitis cases, including 3 of the 5 ST3689 isolates and 2 ST432 isolates.

A single 13/ST156 serotype-switch variant was observed within serotype 13 (sTable 2, category 13).

The lineage of 24F/CC230 was described in the pre-PCV7 years and potentially originated from within a serotype 14 lineage (Pantosti et al., 2002). Serotype 24F included a single 24F/ST177 serotype-switch variant (sTable 2, category 25).

The PCV13 serotypes 9V, 23F, and 14 cumulatively accounted for 36% of ABCs isolates (1442/4046 total isolates, including 477/1044 (46%) pediatric isolates) recovered during 1999 from a surveillance population of about 18.6 million people (Kim et al., 2016). During 2015–2016 isolates of these 3 serotypes were rare (23 total, sFigures 14–16), with no pediatric isolates of these serotypes recovered. The most common serotype during the pre-PCV7 period was serotype 14, which accounted for approximately one third of IPD in children < 5 yrs. Although there were only 6 total serotype 14 isolates during 2015–2016, these residual CCs were common in the pre-PCV7 era and included CC156 (SLVs ST3819 and ST334), CC13, and CC230 (SLV ST5912). Serotype 9V in the US has primarily consisted of both penR and penS sublineages of CC156 throughout the pre and post PCV periods, reflected by these 2 sublineages accounting for all 9 isolates from 2015-2016. Five of the 8 serotype 23F isolates were of the longstanding and highly resistant CC81 lineage (Wyres et al., 2012).

The 3 nontypeable (NT) isolates consisted of 3 unrelated STs. There was a single penR, eryR ST2011 isolate. ST2011 is linked with 10 NT isolates and 2 serogroup 6 isolates in the global MLST database, all recovered during 1999-2007).

## Discussion

This initial depiction of our first 2 years of WGS-based ABCs strain surveillance provides an encompassing data framework for comparison with future strain surveillance. Here WGS has allowed us a closer glimpse into the active process of strain mixing through recombination. The amount of recombination deduced from simply observing 35B/CC558 markers distributed among non serotype 35B strains suggests a species continually engineering and presenting new experimental strains for “testing” the property of fitness. Here we assume that a basic level of fitness is described by the emergence of a strain in our IPD surveillance to the level of detection. For this to have occurred, we assume that the strain was generated and carried within the upper respiratory reservoir for some period of time. For this reason we view with concern the two 3/ST271 isolates described in this study (a third also recovered during 2018; unpublished data). This highly resistant strain combines the current most successful invasive serotype with the most successful invasive clonal complex of the decade before PCV13 implementation (Beall et al., 2011). At this point in time only a minority of newly recognized strain complexes have successfully emerged from these recombinational “experiments” to become detected within multiple ABCs cases. The current IPD burden imposed by the recombinant strains revealed in this study is significant, however our study is limited in detection of such events by the use of recognizable typing patterns gleaned from short-read sequence and incomplete genome assemblies. Increased carriage of serotype 35B by children has been observed in post-PCV years (Sharma et al., 2013; Desai et al., 2015; Kaur et al., 2016). Performing a screen for typing elements consistent with 35B/CC558 within non-35B/CC558 strains allowed us to qualitatively assess the impact of this single common strain complex in recombination events.

Although most of the serotype-switch classes that we have found among these 2015–2016 ABCs isolates were represented by single recombinant strains, there is longstanding evidence that certain serotype-switch “experiments” generate highly adapted and successful strains. While we depict several putative vaccine-escape recombinant strain complexes (e.g., 23B/ST1373, 15A/ST3811, 15BC/ST3820, and 35B/ST156) that have only recently shown successful emergence, serotype switching has been a key mechanism in shaping the pneumococcal population structure long before PCV introduction (Coffey et al., 1991; Wyres et al., 2013). These events often are reflective of recipient and/or donor parental strains that have been known to be abundant within the carriage reservoir, and have often involved gene replacement events that included the *cps* and nearby resistance-conferring *pbp1a* and *pbp2x* alleles (Coffey et al., 1991; Brueggemann et al., 2007; Chochua et al., 2017).

Increasing resistance to beta lactams has been shown to be conducive to the acquisition of additional resistance features, and dually resistant (to penicillin and erythromycin) strains have emerged faster than strains that are resistant to one of these antibiotics alone (Jacobs et al., 1978; McCormick et al., 2003). Within our recent strain surveillance, the emergence of the penI, eryR, cliR 23A/ST338-3 clade has outpaced the eryS, cliS 23A/ST338-1 clade that expresses a lower penicillin MIC (unpublished data). The clade 3/ST180-4 is another example of an emergent offshoot that expresses higher resistance to beta lactams and macrolides than the ancestral clonal complex.

Although many, and possibly all, of the 57 putative recent serotype-switch variants described in this paper may have originated in the pre-PCV era, they were only detected after PCV implementation. Certain of these hybrid strains, as well as older well-documented strains, warrant close scrutiny. Preferential increase or persistence of a specific PCV13 lineage could be consistent with some sort of localized immunologic or epidemiologic advantage. For example, the vaccine serotype 4 isolates from 2015 to 2016 surveillance, primarily the putative switch 4/ST10172 genotype and the well-established 4/ST244 genotype, are almost entirely from western surveillance areas (exclusively California for ST244, Colorado and New Mexico for the ST10172; data not shown) and each genotype constitutes a distinct highly related phylogenetic cluster (sFigure 8). It is striking that 43% of these isolates were from homeless individuals (unpublished ABCs data).

For unknown reasons, pan-susceptible serotype 19F/CC251 strains have declined more slowly than other 19F CCs that were abundant before PCVs and actually increased in IPD slightly in 2016 relative to 2015. It might prove important to examine such strains in PCV13-based opsonophagocytosis assays to evaluate whether antibodies generated in response to type 19F antigen in the vaccine have functionality against these specific type 19F strains. Serotype 6C is an example where such investigation proved important. Originally mistyped as serotype 6A, 6C proved to be a distinct serotype not targeted by PCV7 that differed within its primary repeating unit structure (Park et al., 2007, 2008). Conversely, continued decreases shown within the major 19A CCs is consistent with uniform targeting of these strains by PCV13. It might be relevant to test next generation PCVs for effective targeting of clonal complexes that have recently emerged. For example, although CC199/15BC has been predominant within the U.S. for the past 20 years, it might be prudent to assess the rapidly expanding antimicrobial-resistant 15BC/ST3280 (Andam et al., 2017) for its behavior in next generation PCV-based opsonophagocytic killing assays.

Our primary objective was to provide a meaningful descriptive account of IPD isolates recovered through population-based surveillance in the post-PCV13 era. We hope that these strain data will support a wide array of studies to assist in prevention efforts and contribute to our basic understanding of IPD strains.

## Author's note

The findings and conclusions in this report are those of the authors and do not necessarily represent the official position of the Centers for Disease Control and Prevention.

## Author contributions

All authors reviewed the manuscript and provided constructive feedback. BB wrote the study, performed many of the analyses provided, and guided bioinformatics pipeline development. SC performed, directed, and evaluated all whole genome sequencing data. BM developed the bioinformatics pipeline and provided periodic updates. YL provided the bioinformatics approach for prediction of β-lactam MICs conferred by newly encountered PBP types. ZL, TT, and HW performed whole genome sequencing. LM oversaw all laboratory operations. JR provided phylogenetic analysis for selected serotypes. RG maintained typing antisera as well as performed conventional testing of selected isolates. TP assisted in the writing and provided population-based disease rates for the ABCs data.

### Conflict of interest statement

The authors declare that the research was conducted in the absence of any commercial or financial relationships that could be construed as a potential conflict of interest.

## Supplementary material

sFigures 1–17 Description for Figures 5A–P applies also for these supplementary figures.

sFigures 18-25 General features: color coded rings 1–5 (inner = 1, outer = 5).

1. multilocus sequence type.
2. PBP type.
3. penicillin MICs (μg/ml): gray, 0.06; yellow, 0.12–1; red, >2.
4. erythromycin: gray, susceptible; red, resistant.
5. clindamycin: gray, susceptible; red, resistant.

sFigure 18 Phylogenic resolution of serotype 3 isolates. The 2 major CC180 clades are shown (ST180-2 and ST180-8). ST180-4 corresponds to the indicated eryR, cliR isolates in Figure 5A that also have reduced penicillin-susceptibility (PBP type 2-0-111). The serotype switch variants (CC100 and ST62) are indicated. The two ST271 isolates are not shown since isolates that had under 90% coverage of the reference sequence were excluded from the analysis.

sFigure 19 22F. Phylogenetic resolution of serotype 22F isolates (refer to Figure 5B). Note the separation of clades ST433 (blue) and ST7314 (red-violet, corresponding to eryR isolates). A separate node (ST433-2) of eryR isolates is also indicated.

sFigure 20 Resolution of serotypes 23A and 23B. The separation of CC338 into separate 23A/ST338-1, 23A/ST338-3, and 23B/ST1373 nodes is shown (refer to Figure 5C). Three 23B/ST338-1 switch variants are indicated with asterisks. Note the shorter branch lengths for ST338-3 and ST1373 compared to putatively older clades such as ST338-1.

sFigure 21 Phylogenetic resolution of serotype 12F isolates (corresponds to Figure 5F).

sFigure 22 Resolution of 33F showing the relatively short branch lengths within the ST2705-focused clade compared to the ST100 clade (refer to Figure 5H). In addition, the very high relatedness of the 5 penicillin nonsusceptible ST11856 isolates within CC100 is consistent with very recent emergence.

sFigure 23 11A. Resolution of serotype 11A isolates. The separation of most ST62 (PI-2 negative, primarily associated with PBP type 2-6-10) and ST62-1 (PI-2 positive, primarily associated with PBP type 2-6-0) is indicated (see Figure 5M).

sFigure 24 Resolution of serotypes 15B and 15C isolates. Note the emergent and penI, eryR ST3280 complex. Also consistent with recent emergence are the short branch lengths associated with ST162 (CC156) compared to CC199.

sFigure 25 Serotype 4 resolution. Note the recently emerged ST10172 putative serotype switch variant (refer to and sTable 2 category 18 and sFigure 6).

sTable 1 Information associated with isolate identifiers, including patient ages, sequence quality metrics, bioinformatics pipeline features, and year when recovered.

sTable 2 CCs are listed in order **of decreasing number of progeny isolates**. The MLST/PBP types combinations within each CC are listed such that existing PBP relationships are optimally aligned.
