# Supplementary figures and images for: A Population-Based Descriptive Atlas of Invasive Pneumococcal Strains Recovered Within the U.S. During 2015–2016
Source: Front Microbiol. 2018 Nov 19;9:2670. doi: 10.3389/fmicb.2018.02670 (PMC6262371; doi:10.3389/fmicb.2018.02670)

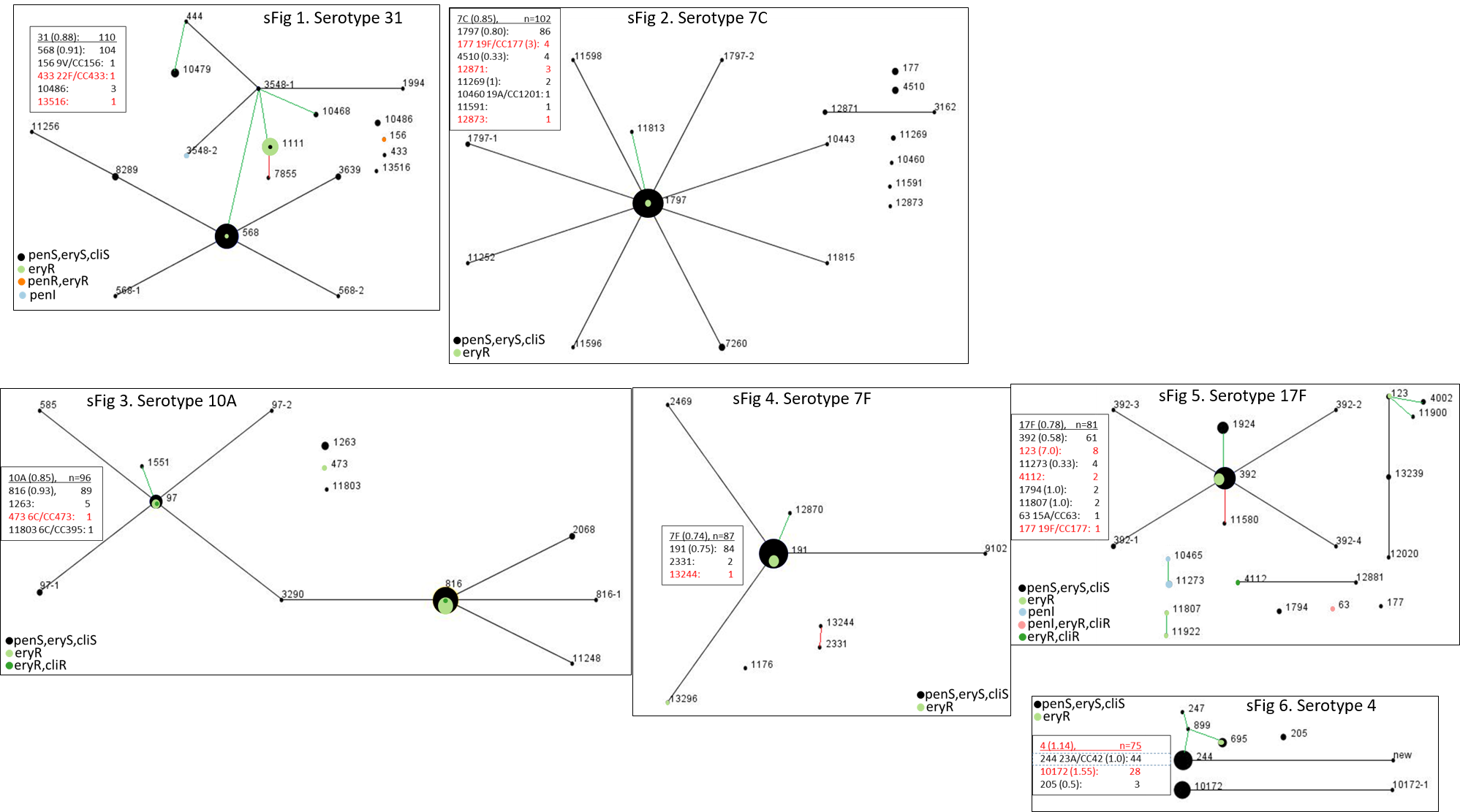

Supplement: Supplementary file 1 [file Image_1.TIF]

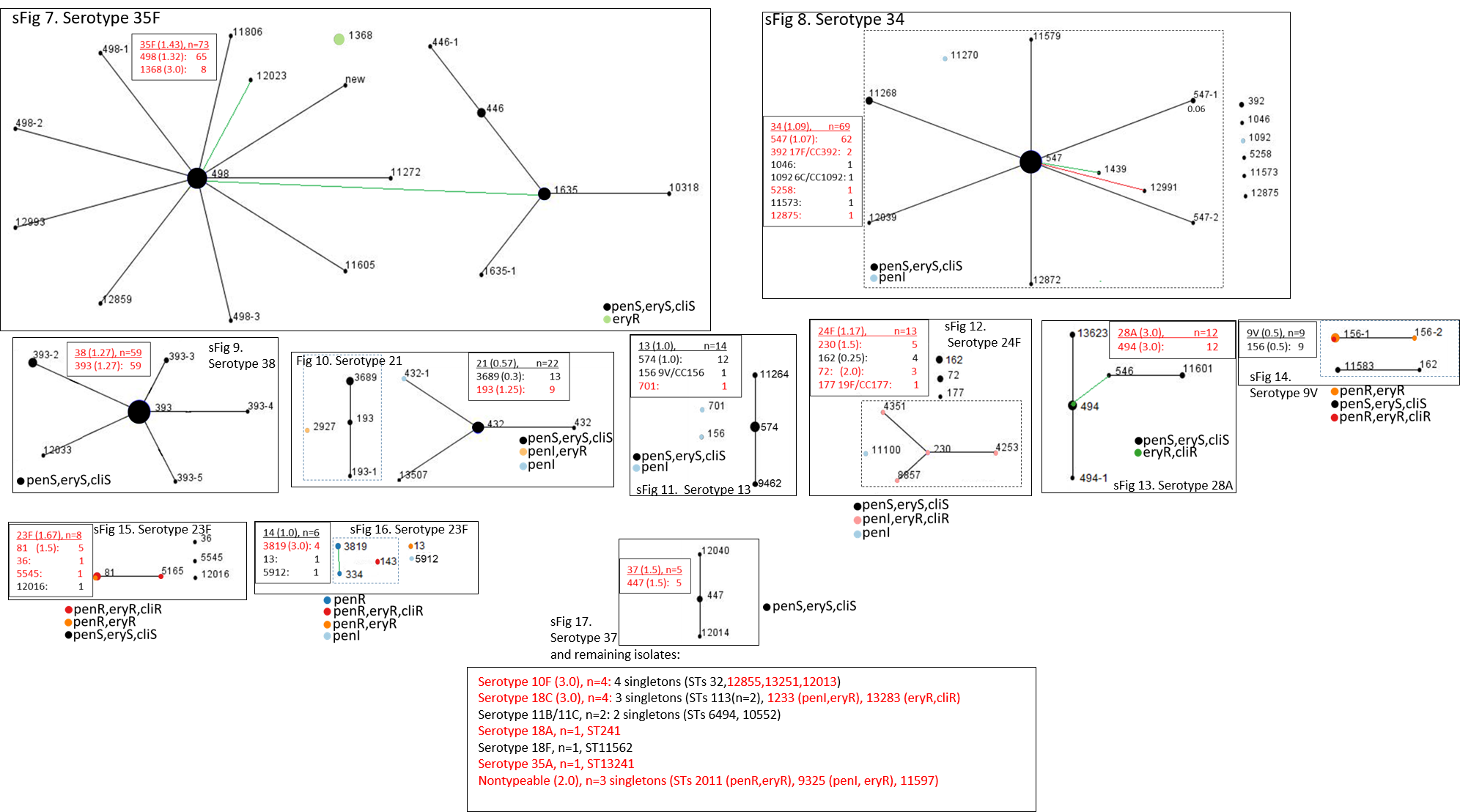

Supplement: sFigures 1–17 — Description for Figures 5A–P applies also for these supplementary figures. [file Image_2.TIF]

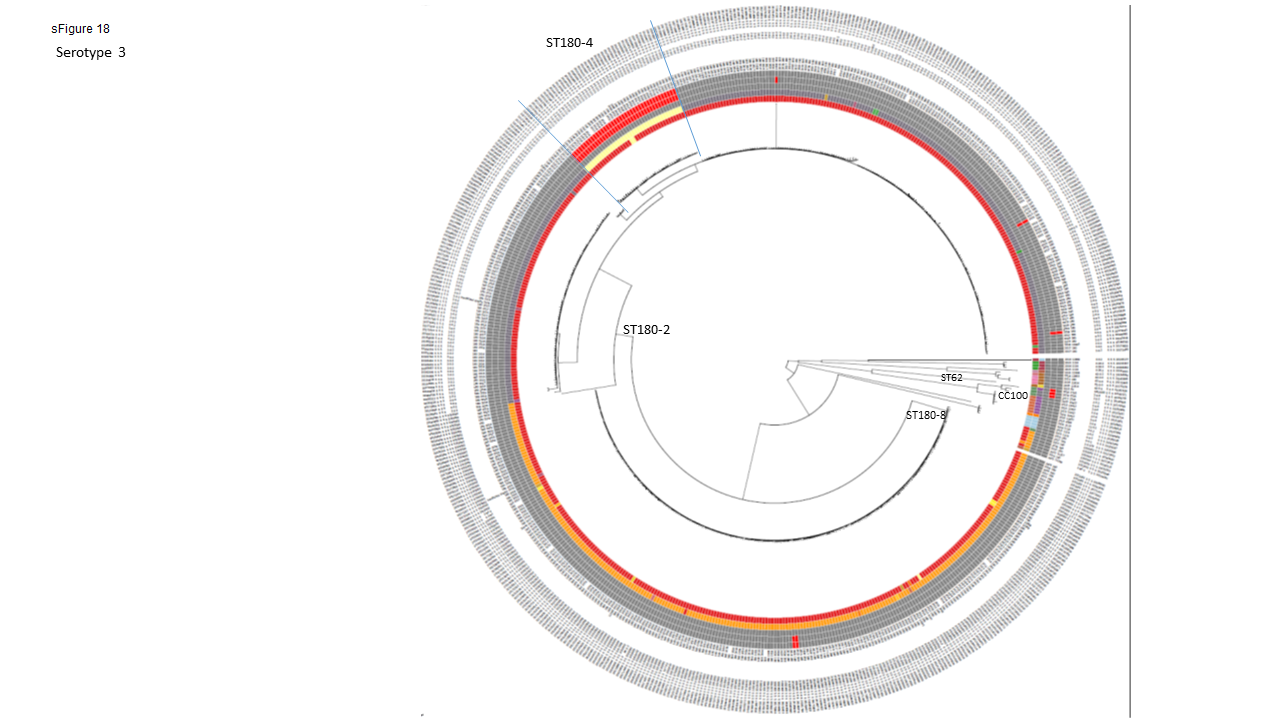

Supplement: sFigures 18-25 — General features: color coded rings 1–5 (inner = 1, outer = 5). [file Image_3.TIF]

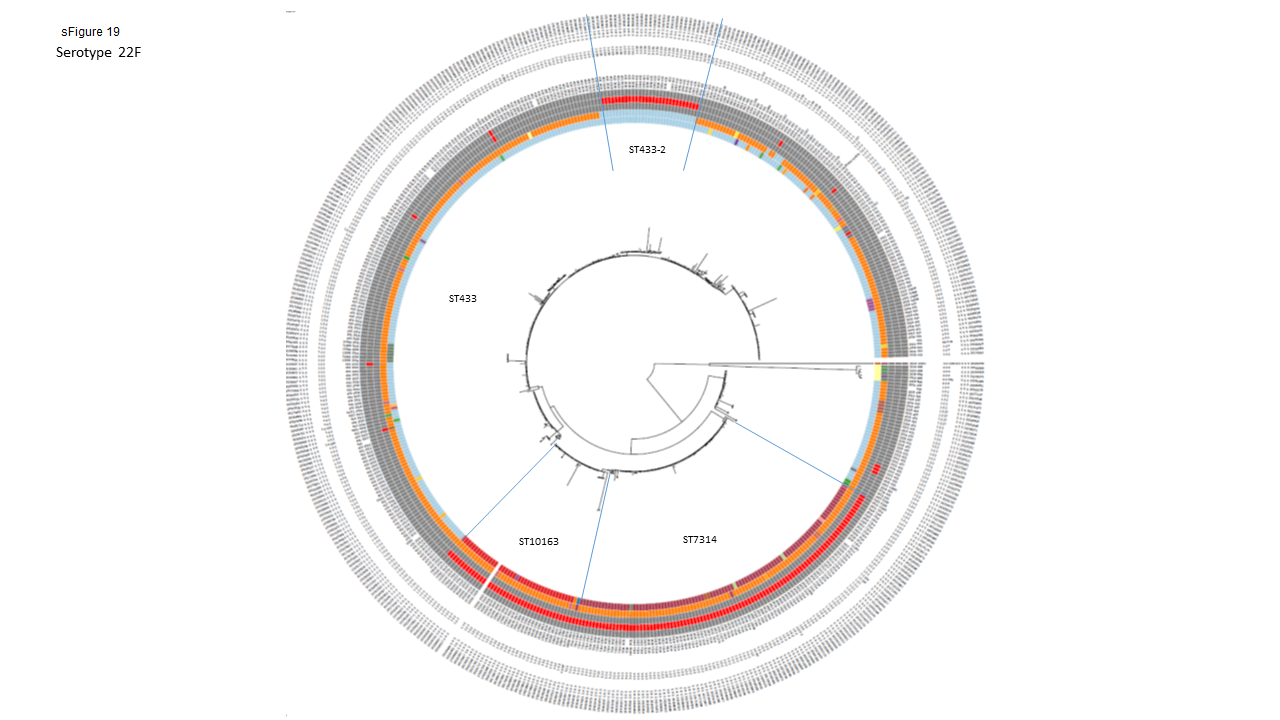

Supplement: sFigure 19 — 22F. Phylogenetic resolution of serotype 22F isolates (refer to Figure 5B). Note the separation of clades ST433 (blue) and ST7314 (red-violet, corresponding to eryR isolates). A separate node (ST433-2) of eryR isolates is also indicated. [file Image_4.TIF]

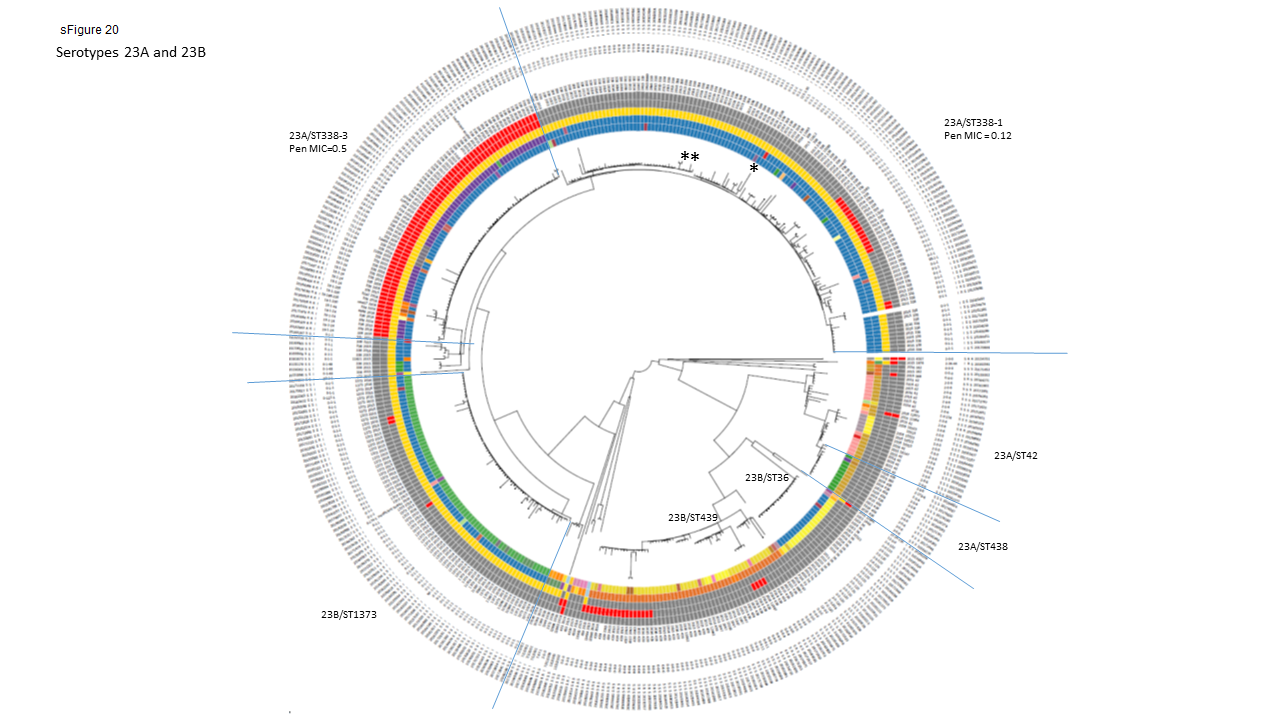

Supplement: sFigure 20 — Resolution of serotypes 23A and 23B. The separation of CC338 into separate 23A/ST338-1, 23A/ST338-3, and 23B/ST1373 nodes is shown (refer to Figure 5C). Three 23B/ST338-1 switch variants are indicated with asterisks. Note the shorter branch lengths for ST338-3 and ST1373 compared to putatively older clades such as ST338-1. [file Image_5.TIF]

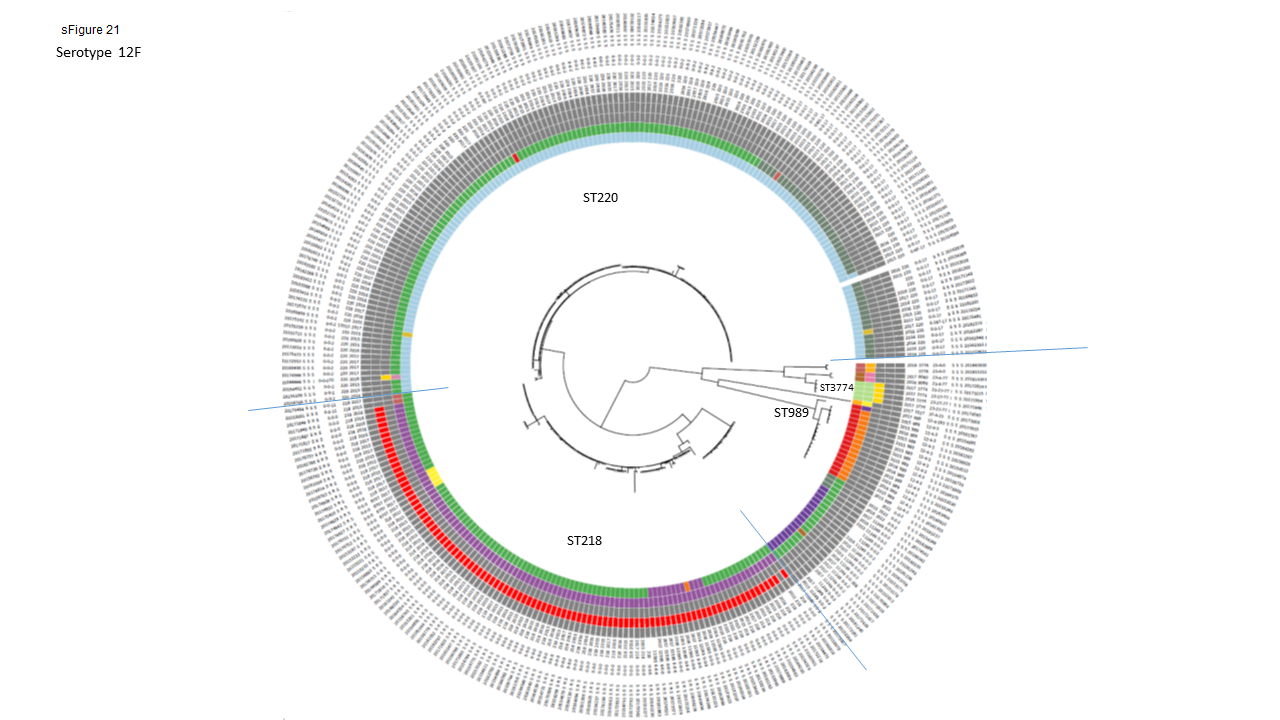

Supplement: sFigure 21 — Phylogenetic resolution of serotype 12F isolates (corresponds to Figure 5F). [file Image_6.TIF]

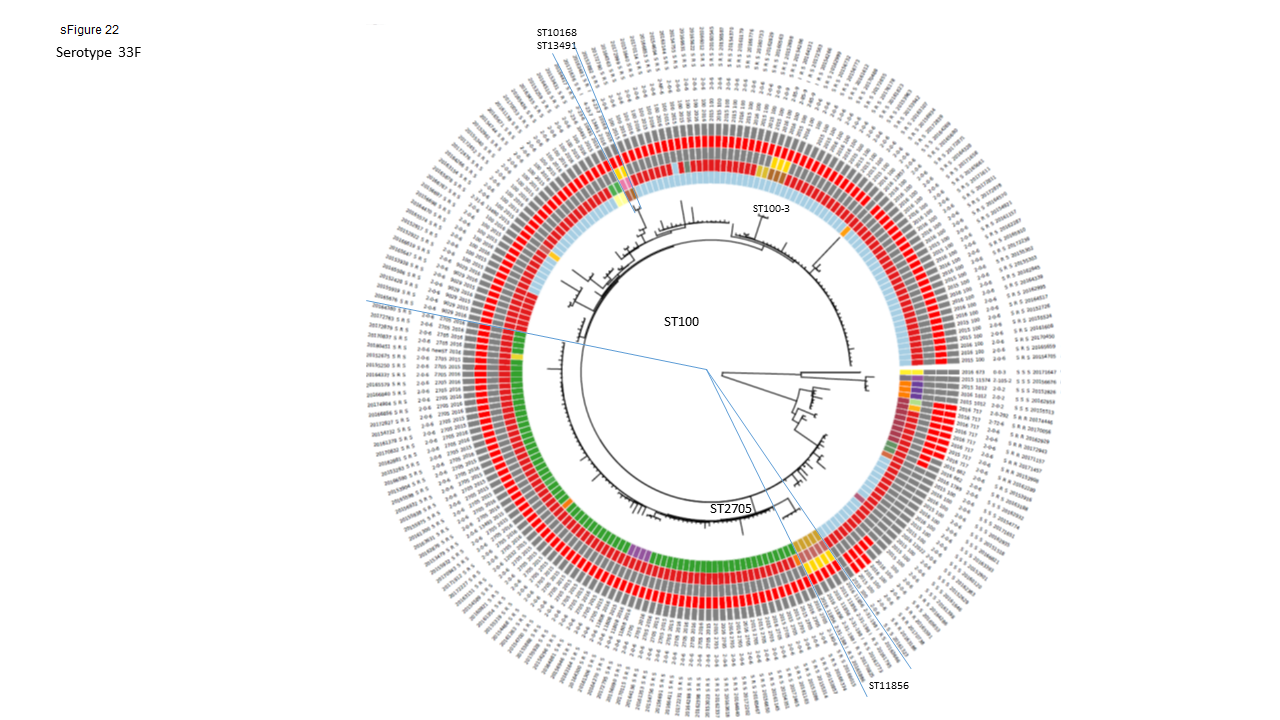

Supplement: sFigure 22 — Resolution of 33F showing the relatively short branch lengths within the ST2705-focused clade compared to the ST100 clade (refer to Figure 5H). In addition, the very high relatedness of the 5 penicillin nonsusceptible ST11856 isolates within CC100 is consistent with very recent emergence. [file Image_7.TIF]

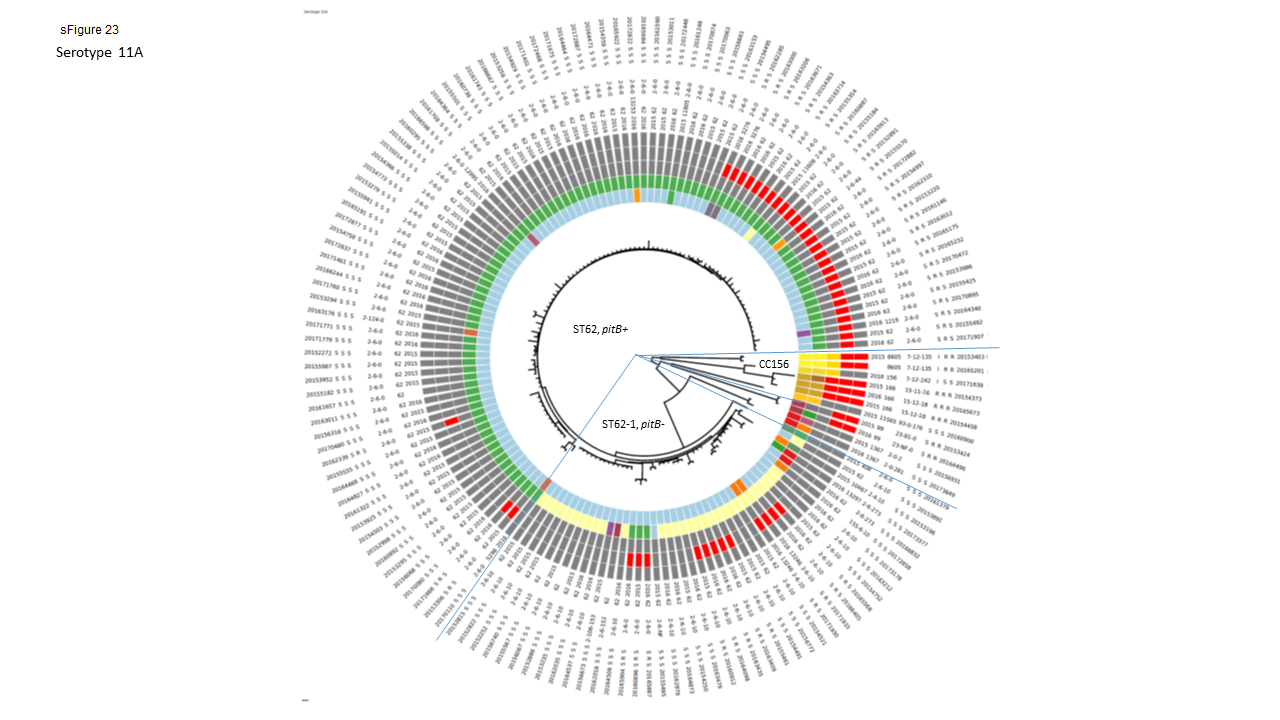

Supplement: sFigure 23 — 11A. Resolution of serotype 11A isolates. The separation of most ST62 (PI-2 negative, primarily associated with PBP type 2-6-10) and ST62-1 (PI-2 positive, primarily associated with PBP type 2-6-0) is indicated (see Figure 5M). [file Image_8.TIF]

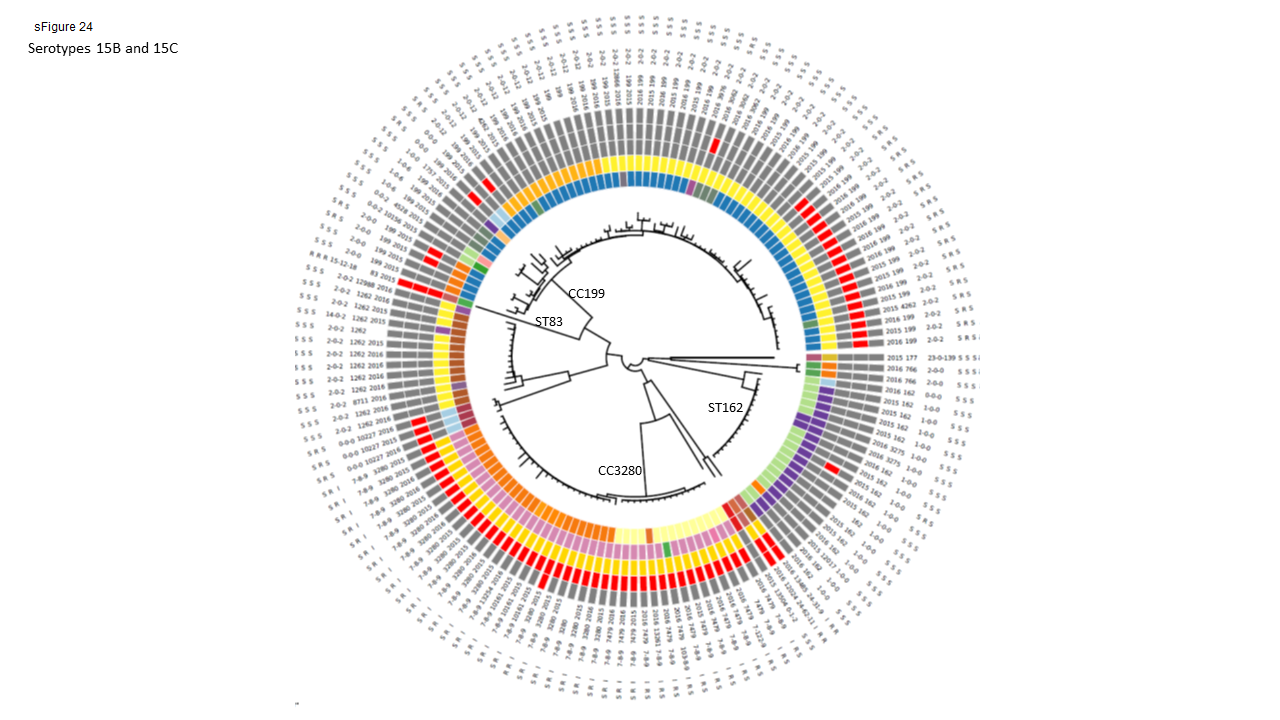

Supplement: sFigure 24 — Resolution of serotypes 15B and 15C isolates. Note the emergent and penI, eryR ST3280 complex. Also consistent with recent emergence are the short branch lengths associated with ST162 (CC156) compared to CC199. [file Image_9.TIF]

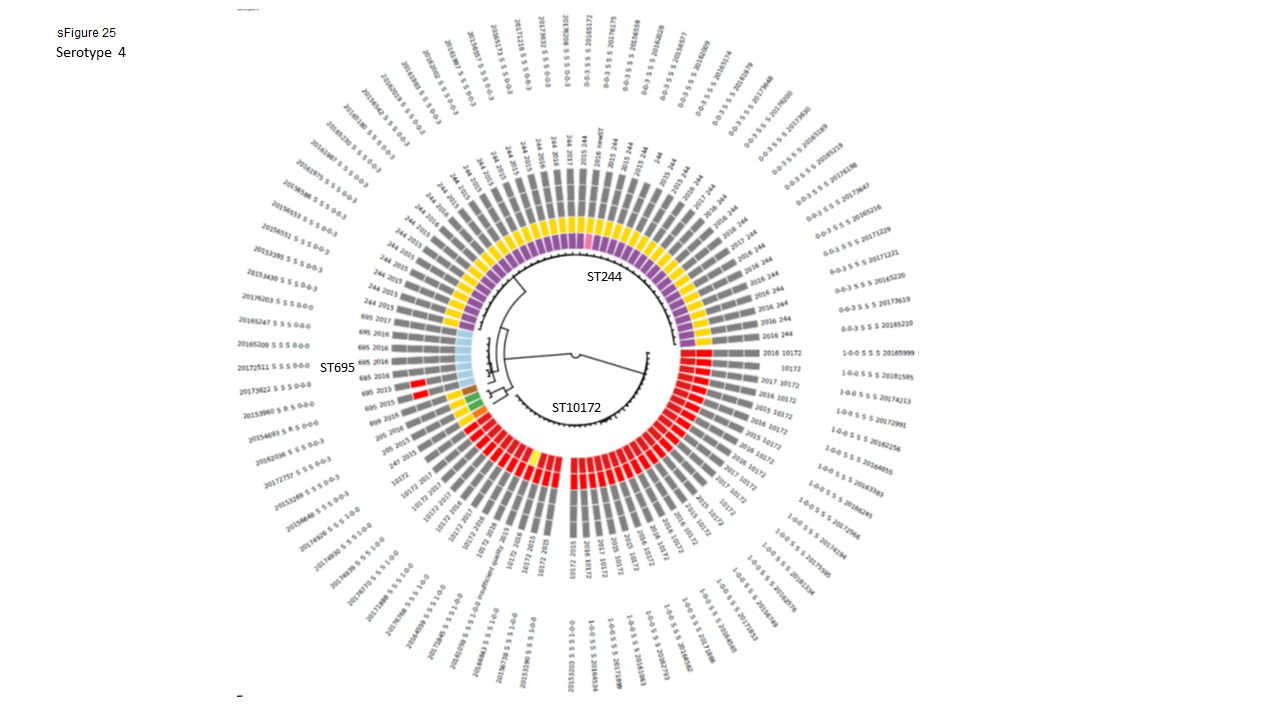

Supplement: sFigure 25 — Serotype 4 resolution. Note the recently emerged ST10172 putative serotype switch variant (refer to and sTable 2 category 18 and sFigure 6). [file Image_10.TIF]
