# Supplementary material for: A Population-Based Descriptive Atlas of Invasive Pneumococcal Strains Recovered Within the U.S. During 2015–2016
Source: Front Microbiol. 2018 Nov 19;9:2670. doi: 10.3389/fmicb.2018.02670 (PMC6262371; doi:10.3389/fmicb.2018.02670)
Supplement: sTable 2 — CCs are listed in order of decreasing number of progeny isolates. The MLST/PBP types combinations within each CC are listed such that existing PBP relationships are optimally aligned. [file Table_2.pdf]

supplementary Table 2. Clonal complex/serotype combinations of serotype-switch variants within 2015-2016 ABCs that have been documented from isolates recovered during 2002 or later.

CC[A]	putative replaced serotype	Progeny serotype	No. progeny isolates	Predominant or representative[A]		pen,ery,cli phenotypes (no. isolates)	PCV escape	PCV13 serotype progeny	category
				MLST	PBP type				
338	23A	23B	101	1373	0-1-1	penI (101)	no	no	1
		19F	1	338	0-1-1	penI, eryR (1)	no	yes	2
		35B	1	13255	0-1-1	penI (1)	no	no	3
		15C	1	13504	0-1-2	susceptible (1)	no	no	4
		15A	1	338	36-1-69	penI (1)	no	no	5
		19A	2	8197	17-1-22	penI (2)	no	yes	6
		6C	3	5241	0-86-80	penI (3)	no	yes	7
156	9V,19A,19F	15A	40	3811	1-0-0	susceptible (39); penI,eryR (1)	yes	no	8
		15BC	22	162	1-0-0	susceptible (21); eryR (1)	yes	no	9
		23B	2	162	0-0-0	susceptible (2)	yes	no	10
		11A	4	166	15-12-18	penR, eryR, cliR (3), penI (1)	yes	no	11
		31	1	156	15-12-18	penR, eryR (1)	yes	no	12
		13	1	156	15-12-173	penI (1)	yes	no	13
		35B	27	156	4-12-7	penR, eryR (26); susceptible (1)	yes	no	14
3280	23F	15BC	59	3280	7-8-9	penI,eryR (58); penI,eryR,cliR (1)	yes	no	15
244	4	19A	48	695	8-0-11	penI (34); penI, eryR (14)	no	yes	16
		19F	1	899	0-0-3	susceptible (1)	no	yes	17
439	23A	4	28	10172	1-0-0	susceptible (28)	no	yes	18
100	33F	3	8	10457	2-0-6	susceptible (6); eryR (2)	no	yes	19
		6C	1	10471	2-0-6	penI (1)	no	yes	20
		23A	1	10492	0-0-1	penI, eryR (1)	no	no	21
177	19F	15C	1	177	23-0-139	susceptible (1)	yes	no	22
		17F	1	177	23-0-0	susceptible (1)	yes	no	23
		7C	4	177	2-0-3	susceptible (4)	yes	no	24
		24F	1	177	1-0-0	susceptible (1)	yes	no	25
63	15A	19F	1	861	24-27-192	penI, eryR, cliR (1)	no	yes	26
		8	1	63	24-27-28	penI, eryR, cliR (1)	no	no	27
		9N	1	2543	1-27-2	eryR, cliR (1)	no	no	28
		17F	1	63	0-27-3	penI, eryR, cliR (1)	no	yes	29
		15BC	2	12024	24-62-11	penI, eryR, cliR (2)	no	no	30
		35B	1	11818	13-31-114	penI, eryR, cliR (1)	no	no	31
		23B	2	12863	7-31-8	penI, eryR, cliR (2)	no	no	32
433	22F	23B	5	433	1-2-0	susceptible (5)	no	no	33
		31	1	433	1-2-0	susceptible (1)	no	no	34
		11A	1	433	1-2-2	susceptible (1)	no	no	35
193	21	15B	3	10227	0-0-0	eryR (3)	no	no	36
		23A	1	3689	0-0-1	penI (1)	no	no	37
376	6A, 6C	19A	3	2268	27-36-8	penR, eryR (3)	no	yes	38
81	19F, 23F	6A	1	282	15-12-18	penR, eryR (1)	no	yes	39
		15A	1	11576	15-12-18	penR, eryR (1)	yes	no	40
		15C	1	83	15-12-18	penR, eryR (1)	yes	no	41
473	6C	10A	1	473	0-34-2	eryR (1)	yes	no	42
		23B	1	1876	2-34-44	penI, eryR (1)	yes	no	43
320	19F	3	2	271	17-16-47	penR, eryR, cliR (2)	no	yes	44
1092	6C	34	1	1092	6-101-8	penI (1)	yes	no	45
		35B	1	1092	6-7-36	penI (1)	yes	no	46
230	19A	20	2	276	17-15-18	penR, eryR, cliR (2)	yes	no	47
62	11A	3	1	62	2-6-0	susceptible (1)	no	yes	48
242	23F	6A	1	242	13-31-146	penR, eryR, cliR (1)	no	yes	49
392	17F	34	1	392	11-0-0	susceptible (1)	no	no	50
395	6C	10A	1	11803	1-6-0	susceptible (1)	yes	no	51
432	21	19A	1	432	0-0-2	susceptible (1)	no	yes	52
504	23F	8	1	504	3-2-5	susceptible (1)	yes	no	53
558	35B	6B	1	558	4-7-7	penR (1)	no	yes	54
568	31	23B	1	568	0-0-0	eryR (1)	no	no	55
1201	19A	7C	1	10460	2-4-3	susceptible (1)	no	yes	56
1292	6C	16F	1	10464	19-31-8	penI, eryR (1)	yes	no	57

[A] CCs (clonal complexes) are listed in order of decreasing number of progeny isolates. The MLST/PBP type combinations within each CC are listed such that existing PBP relationships are optimally aligned.
